# Heterointerface‐Functionalized Photoelectric Response of Metal‐Oxide Schottky Photodiode for Intelligent Fire Detection

**DOI:** 10.1002/advs.202519318

**Published:** 2025-12-03

**Authors:** Yuyang Cai, Zhiwei Zheng, Zhiwu Zhong, Yuhan Zhang, Tengyan Huang, Yucheng Cao, Dawei Zheng, Yen Hung Lin, Fion Sze Yan Yeung, Kuan‐Chang Chang, Jie Chen, Hoi Sing Kwok, Lei LU, Yufeng Jin

**Affiliations:** ^1^ School of Electronic and Computer Engineering Peking University Shenzhen 518055 China; ^2^ State Key Laboratory of Displays and Opto‐Electronics The Hong Kong University of Science and Technology Clear Water Bay Kowloon Hong Kong SAR China

**Keywords:** heterointerface, intelligent photodetection application, low temperature, oxide semiconductor, Schottky photodiode

## Abstract

Ultraviolet photodetectors (UV‐PDs) based on wide‐bandgap oxide semiconductors (OSs) hold broad prospects in scientific, civil, and especially fire alarm applications. However, the hotly pursued flexible and hetero‐integration capabilities are hindered by the high processing temperature of mainstream Ga_2_O_3_ UV‐PDs, while the selective UV detection capability of low‐temperature OS (LT‐OS) is undermined by the pronounced contradictory effects of abundant native defects on electric and optoelectrical properties. Herein, a heterointerface‐functionalized Schottky photodiode (HF‐SPD) is proposed to realize high‐performance LT‐OS UV‐PD. Due to the sophistically modulated Schottky contact and defect distribution, ultralow dark current and remarkable photoresponse are simultaneously achieved in In_2_O_3_ HF‐SPDs. Under the typical 360‐nm UV illumination, the responsivity (*R*) and detectivity (*D* ^*^) respectively reach 27.75 A W^−1^ and 2.036 × 10^13^ Jones, enabling the early‐fire‐warning capability. Moreover, the HF‐SPD possesses unique wavelength selectivity and bias‐tunable photoelectrical characteristics. By feeding such multidimensional feature data into the multi‐layer perceptron (MLP) neural network, the pattern recognition of the spectrum delivers a remarkable accuracy of 99.94%, ensuring the precise identification of combustion materials. Such an intelligent fire warning system demonstrates the prospects of algorithm‐boosted LT‐OS HF‐SPDs in ubiquitous intelligent systems, such as wearable photodetection and in‐sensor computing (ISC) devices.

## Graphical abstract

A heterointerface‐functionalized Schottky photodiode (HF‐SPD) is developed to explore the photoelectric capabilities of low‐temperature oxide semiconductors. Besides the remarkable UV detection capability, the bias‐tunable photo response endows HF‐SPD with the unique wavelength selectivity. Integrated with an MLP neural network, it attains 99.94% accuracy in burning material identification, demonstrating the potential of low‐temperature HF‐SPD for wearable photo‐sensing and in‐sensor computing applications.

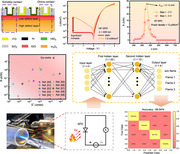

## 1 Introduction

The UV photodetectors (PDs) play irreplaceable roles in extensive fields, including solar astronomy,^[^ ^1^, ^2^ ^]^ ozone layer surveillance,^[^ ^3^ ^]^ optical communication,^[^ ^4^, ^5^ ^]^ combustion monitoring,^[^ ^6^ ^]^ and wearable healthcare monitoring.^[^ ^7^ ^]^ Particularly for frequent fire disasters, effective firefighting critically depends on the rapid and precise identification of combustion materials,^[^ ^8^, ^9^ ^]^ necessitating optoelectronic detection systems capable of sensitively extracting and intelligently analyzing the multidimensional features of flame UV spectra.^[^ ^10^, ^11^ ^]^ The sensitive UV acquisition necessitates both the strategic selection of wide‐bandgap (WBG) semiconductors and the deliberate design of device architectures.^[^ ^12^, ^13^ ^]^ As a representative WBG oxide semiconductor (OS), single‐crystal gallium oxide (Ga_2_O_3_) bestows low dark current and high visible‐blind photoresponse on mainstream UV‐PDs.^[^ ^14^, ^15^ ^]^ However, the high growth temperature (>1200 °C) of Ga_2_O_3_ prohibits their applications in flexible optoelectronics and emerging in‐sensor computing (ISC) systems.^[^ ^16^ ^,^ ^17^ ^]^

Building upon the remarkable success of WBG amorphous indium‐gallium‐zinc oxide (a‐IGZO) field‐effect transistors (FETs) in the display industry,^[^ ^18^, ^19^, ^20^ ^]^ significant research attention has been directed toward flexible electronics and heterointegration applications utilizing these low‐temperature OSs (LT‐OSs, typically processed below 500 °C).^[^ ^21^, ^22^ ^]^ Correspondingly, LT‐OS PDs have been largely developed by equipping three‐terminal FETs with photoconductive channels. The LT‐OS channels are either populated with visible‐light‐sensitive oxygen vacancy (Vo) states^[^ ^23^, ^24^ ^]^ or hetero‐integrated with UV‐sensitive materials, such as 2D^[^ ^25^ ^]^ and halide perovskite semiconductors,^[^ ^26^ ^]^ while the reliable discrimination of UV spectra urges more advanced strategies beyond the photoconductive channel.

Among various architectures of WBG semiconductor UV‐PDs, the photo‐tunable Schottky barrier endows the simple two‐terminal Schottky photodiode (SPD) with ultralow dark current and high photoelectric gain.^[^ ^27^, ^28^, ^29^ ^]^ However, abundant Vo and other native defects root in the weak In‐O bonds of LT‐OSs and inevitably induce the severe Fermi pinning effect at the defective Schottky interface,^[^ ^30^ ^]^ leading to the undesirably high dark current. In contrast, the incorporation of stronger Ga‐O bonds effectively suppresses these oxygen‐related defects, but meanwhile enlarges the bandgap of LT‐OSs,^[^ ^31^ ^]^ delivering photo‐insensitive LT‐OSs.^[^ ^18^ ^]^ Such inherent contradictory effects on electric and optoelectrical properties critically undermine the development of LT‐OS SPDs.

Herein, a novel heterointerface‐functionalized SPD (HF‐SPD) is proposed to fundamentally overcome this long‑standing trade‑off by strategically modulating the spatial distribution of Vo in the LT‐OS bulk and at the Schottky interface. Based on the synergistic effects of photo‐triggered Vo ionization and field‐assisted carrier tunnelling, HF‐SPDs realize the distinctive multidimensional photoresponse with wavelength selectivity and bias tunability. Integrated with neural network algorithms, the device enables instantaneous and accurate classification of combustion materials, inspiring the prospects for early fire warning and intelligent spectral discrimination. Such heterointerface‐functionalized methodology maximizes the exploitation of LT‐OS material diversity to enable the versatile flexible and hetero‐integration optoelectronic systems.

## 2 Results and Discussion

### 2.1 Heterointerface Engineering Strategy

**Figure 1:**
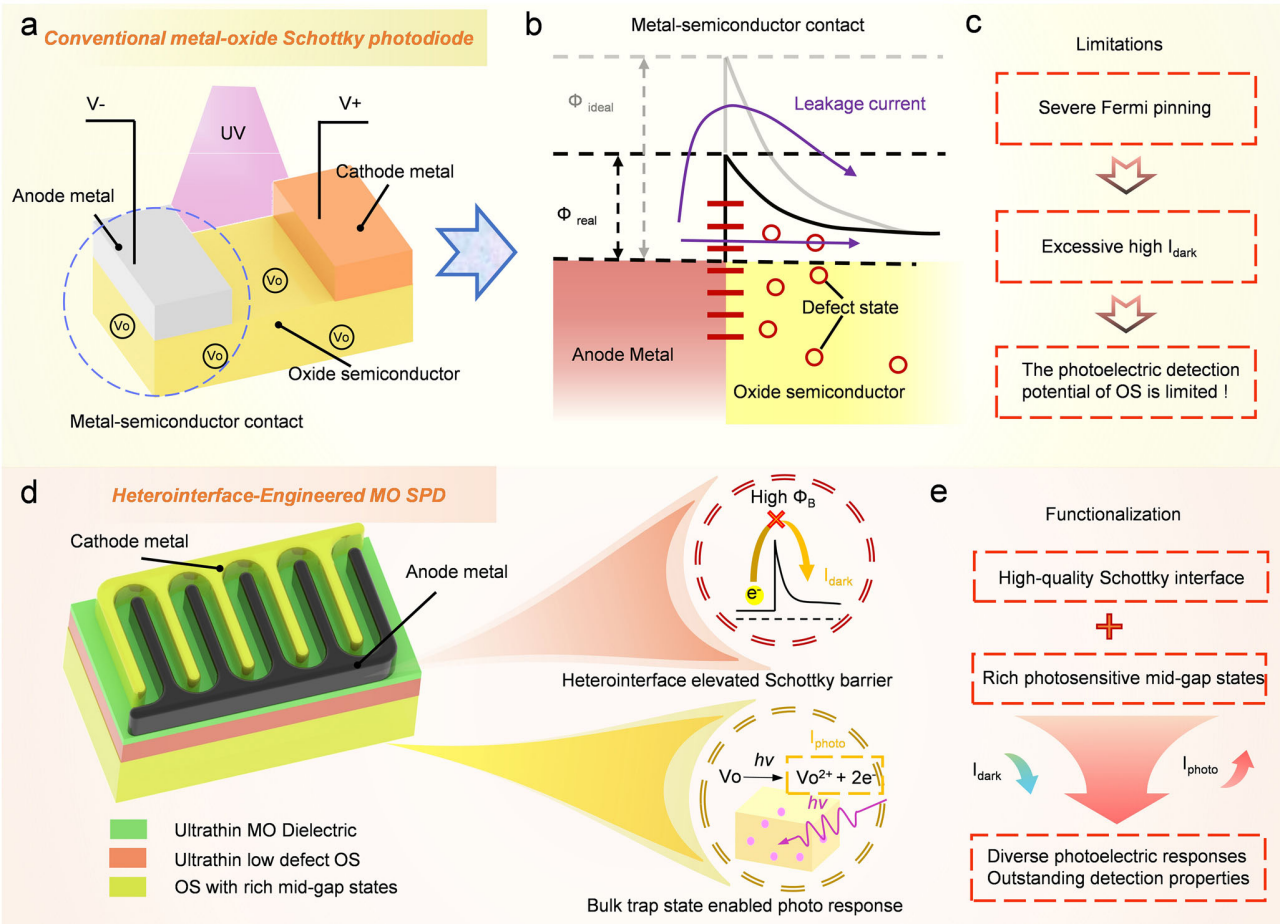
Schematic illustration of the interface engineering strategy for OS SPD. Conventional OS SPD: a) Device schematic, b) defect‐induced Fermi pinning at Schottky interface, c) and limitations on photodetection. Novel MO HF‐SPD: d) Device schematic with insets suggesting the effects of heterointerface and bulk, e) and functionalization mechanism of photodetection.

Indium is essential for supplying free carriers in LT‐OSs. At the same time, In‐O bonds are relatively weaker than Ga‐O bonds and thus give rise to abundant native Vo defects, since the oxygen deficiency in metal‐oxide (MO) materials is directly governed by the strength of M–O bonds.^[^ ^32^ ^]^ In an LT‐OS SPD (**Figure** **1**a,b), ubiquitous Vo defects readily cause severe Fermi‐level pinning at the Schottky contact interface. This leads to a significant reduction in barrier height and an excessively high dark leakage current (*I*_dark_) under reverse bias, fundamentally limiting the photodetection performance (Figure 1c).

As illustrated in Figure 1d, a heterointerface engineering strategy is developed to impart UV detection capability to MO Schottky barrier diodes (SBDs). The anode/OS contact is adapted with ultrathin M‐O interlayers featuring stronger M‐O bonds, such as Hf‐O and Ga‐O. The interface oxygen deficiency and corresponding Fermi level pinning are thus effectively mitigated. Such an elevated Schottky barrier height (*Φ*_B_) effectively reduces *I*_dark_, while mid‐gap states in the OS bulk facilitate the photoexcitation of carriers. Such heterointerface‐functionalized SPD (HF‐SPD) can be implemented by synergistically utilizing the precise atomic layer deposition (ALD) and the high‐efficiency sputtering. Such a low‐defect interface and photosensitive bulk enable tunable photoelectric responses and outstanding detection sensitivity, as illustrated in Figure 1e.

### 2.2 Fabrication and Characterization of In_2_O_3_ HF Schottky Barrier Diodes

**Figure 2:**
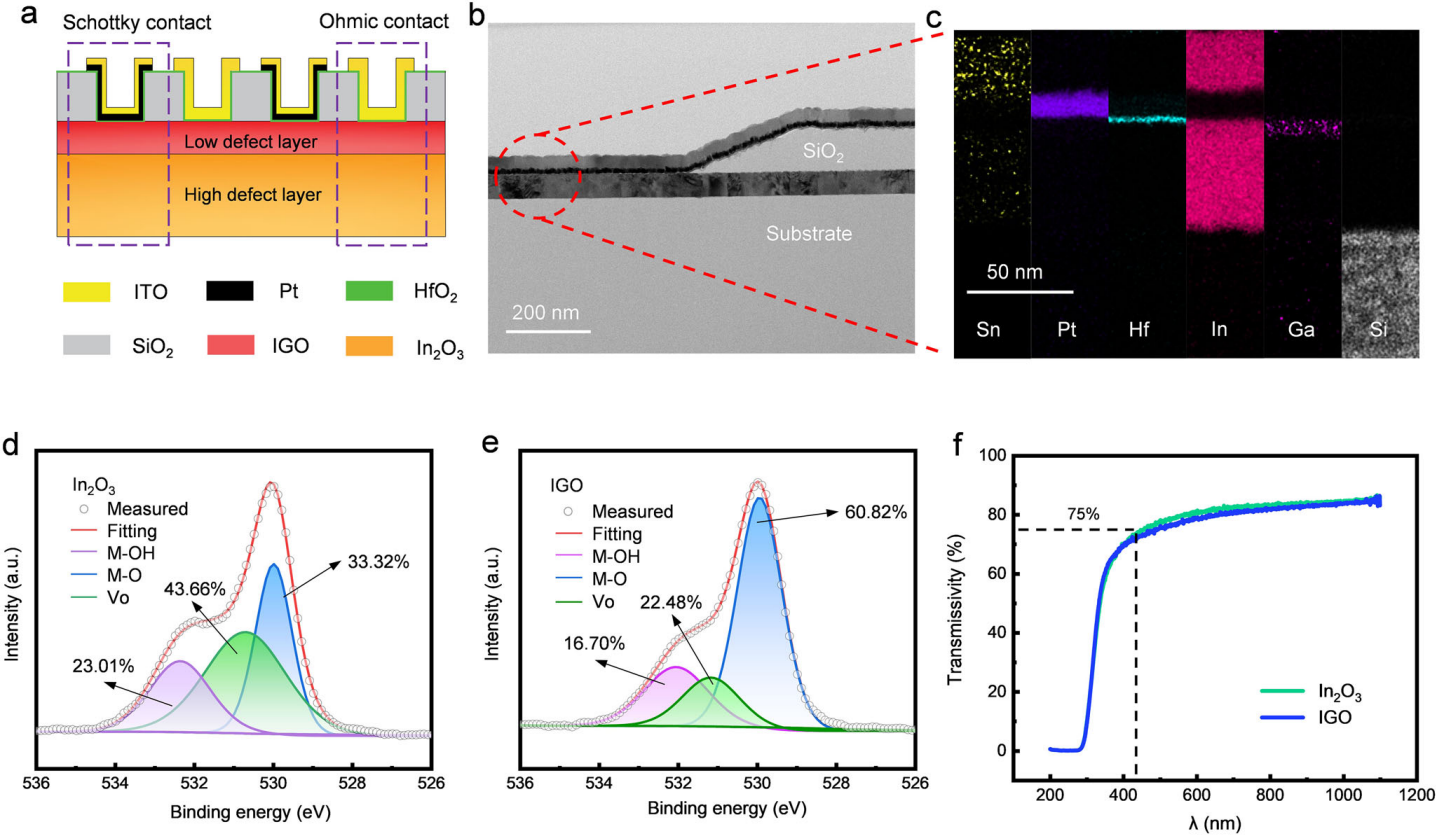
Structural and material analyses of heterointerface‐engineered In_2_O_3_ SBD: a) schematic cross‐section, b) cross‐sectional TEM morphology, c) EDS elemental profiles, d,e) XPS spectra of O s1 and f) optical transmittance spectra of In_2_O_3_ and IGO.

As shown in **Figure** **2**a, a 40‐nm In_2_O_3_ film was first sputtered on the substrate. A 100‐nm‐thick SiO_2_ passivation layer (PL) was then deposited via plasma‐enhanced chemical vapor deposition (PECVD) and patterned to expose the contact areas for electrodes. Thermal oxidation was performed at 400 °C to repair the etching damage. Subsequently, a 2‐nm ALD HfO_2_ film was deposited as the defect‐inhibiting interlayer. Platinum (Pt) and indium‐tin oxide (ITO) were sputtered and patterned to serve as anode and cathode, respectively. For some devices, an 8‐nm indium‐gallium oxide (IGO) film is further inserted between In_2_O_3_ and HfO_2_. As evaluated by ultraviolet photoelectron spectroscopy (UPS), the large work function (W_F_) difference between OSs and Pt tends to form high Schottky barriers (Figure S1, Supporting Information), while low‐W_F_ ITO forms the ohmic contact cathode. The detailed fabrication process is presented in Figure S2 (Supporting Information). Such an LT‐OS SBD possesses promising potential for flexible electronics and heterogeneous integration applications.

Figure S3 (Supporting Information) shows the scanning electron microscope (SEM) image of the resulting heterointerface‐engineered In_2_O_3_ SBD. The interdigitated electrodes of Pt and ITO are effectively isolated, corresponding to Schottky and ohmic contacts, respectively. Focused ion beam (FIB) sectioning and transmission electron microscope (TEM) analysis reveal the sharp Schottky interface, as shown in Figure 2b. Energy‐dispersive spectroscopy (EDS) mapping (Figure 2c) further shows the clear stratification of the heterojunction, confirming the absence of observable element interdiffusion during thermal treatments. In comparison, the direct contact between In_2_O_3_ and Pt is observed in the conventional In_2_O_3_ SBD (Figures S4 and S5, Supporting Information).

X‐ray photoelectron spectroscopy (XPS) was performed to semi‐quantitatively compare the oxygen deficiencies of In_2_O_3_ and IGO.^[^ ^33^ ^]^ The comparison of O 1s spectra in Figure 2d,e reveals a much higher Vo content in In_2_O_3_ is than that in IGO, since the Ga‐O bonds are much stronger than In‐O bonds and thus effectively inhibit oxygen deficiency and electron concentration in LT‐OSs, which is also confirmed by XPS spectra of In 3d in Figure S6 (Supporting Information). Therefore, an 8 nm IGO interlayer can mitigate the Schottky interface defects and thus improve the In_2_O_3_ SBD.^[^ ^31^ ^]^ However, the enhanced current rectification ratio is still lower than 90, as shown in Figure S7 (Supporting information). Most plausibly, the electron wave function of anode metal may penetrate into the OS interior, triggering the metal‐induced gap state (MIGS) and accordingly lowering the Schottky barrier. As suggested by our investigations^[^ ^34^ ^]^ and other reports,^[^ ^35^, ^36^ ^]^ an ultrathin insulating interlayer may be able to attenuate MIGS at the Schottky contact. Thus, 2‐nm HfO_2_ with strong Hf‐O bond and a high dielectric constant is incorporated between OS and Pt. Its effectiveness on Fermi level de‐pinning is confirmed by the significantly elevated current rectification ratio of 3.34 × 10^4^, as detailly described in Note S1 and Figure S7 (Supporting information).

As shown in Figure 2f, In_2_O_3_ and IGO exhibit similar optical transmission spectra. The high visible light transmittance dramatically drops with the wavelength (*λ*) decreasing to below 430 nm, suggesting the potential for ultraviolet and near‐ultraviolet detection. The Tauc plotting method is utilized to extract the bandgap width. The relationship between absorption coefficient and optical bandgap (*E*_g_) is illustrated by the following expression:^[^ ^37^ ^]^

(1) $${\left(\frac{t}{lnT^{-1}}h\nu \right)}^{\frac{1}{2}}=A\left(h\nu -E_{g}\right)$$

where *hν*, *t* and *T* represent the energy of the incident photon, the thickness of the film, and the optical transmittance, respectively. From the ${\left(\frac{t}{\ln T^{-1}}h\nu \right)}^{\frac{1}{2}}$‐*h*ν curves (Figure S8, Supporting Information), *E*_g_ is extracted to be 3.54 and 3.63 eV respectively for In_2_O_3_ and IGO, corresponding to band‐to‐band UV (*λ* < 350 nm) photon excitation. The partial absorption of violet light (*λ* = 350 nm ∼ 430 nm) is normally ascribed to the assistance of mid‐gap states, such as deep‐state Vo.^[^ ^38^ ^]^ It is noteworthy that the photo‐ionization of neutral Vo into ionized Vo (Vo^2+^) is the foundation of incumbent LT‐OS phototransistors.^[^ ^39^ ^]^

### 2.3 Comparison of In_2_O_3_ SPDs in Conventional and Heterointerface Structures

**Figure 3:**
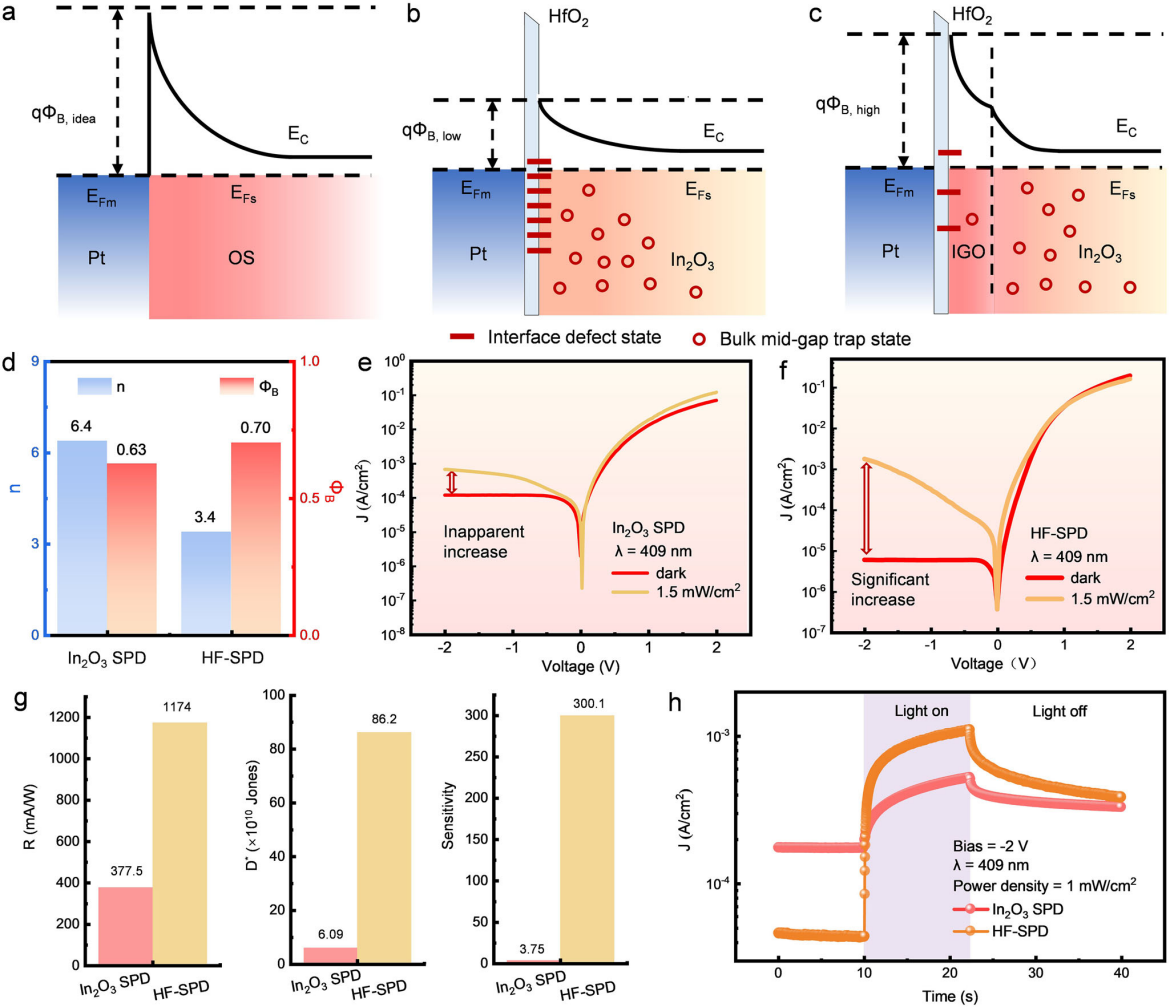
Comparison of band diagrams and photoelectric properties of In_2_O_3_ SPDs in conventional and heterointerface structures. Band diagrams of a) ideal, b) defect‐deteriorated, and c) heterointerface‐enhanced Schottky contacts. d) *n* and *Φ*_B_ of In_2_O_3_ SPD and HF‐SPD. Dark and illuminated *I–V* characteristics of e) In_2_O_3_ SPD and f) In_2_O_3_ HF‐SPD. g) Comparisons on responsivity, detectivity, and sensitivity of In_2_O_3_ SPD and HF‐SPD subjected to 409‐nm illumination at an optical power density of 1.5 mW cm^−2^. h) Dynamic photoelectric responses of In_2_O_3_ SPD and HF‐SPD.

The heterointerface engineering methodology for In_2_O_3_ SBD is illustrated in **Figure** **3**. The interface between oxygen‐deficient In_2_O_3_ and oxidation‐resisting Pt is inherently populated with Vo defects, resulting in Fermi level pinning and thus lowering *Φ*_B_ (Figure 3b). The ultrathin low‐defect IGO interlayer is introduced to improve the Schottky barrier, while these photosensitive mid‐gap states are maintained in the In_2_O_3_ bulk (Figure 3c).

The effects of such heterointerface engineering on dark *I–V* characteristics of In_2_O_3_ SPDs are demonstrated in Figure S9 (Supporting Information), including a higher rectification ratio and uniformity. As detailed in Note S2 and Figure S10 (Supporting Information), the Cheung method was utilized to extract basic parameters of Schottky diodes.^[^ ^40^ ^]^ As compared in Figure 3d, the considerably poor ideal factor (*n*) of 6.4 and low *Φ*_B_ of 0.63 eV for In_2_O_3_ SPD suggest noticeable nonideal mechanism besides the basic thermal emission current, such as the interface state‐assisted tunneling through the ultrathin HfO_2_ interlayer. For HF‐SPDs with ultrathin IGO further mitigating interface defects,^[^ ^31^ ^]^ *n* and *Φ*_B_ are significantly improved to 3.4 and 0.7 eV, together with noticeably lowered *I*_dark_. As verified in Figure S11 (Supporting Information), such high‐performance metrics can be long‐term maintained in the atmospheric environment due to the optimized passivation layer.^[^ ^41^ ^]^ These achievements pave the way for OS SBDs in photodetection applications.

The photoelectric characteristics of In_2_O_3_ SPDs and HF‐SPDs were evaluated with a wavelength of 409 nm and an optical power density of 1.5 mW cm^−2^. Whereas the In_2_O_3_ SPD shows only a negligible increase in reverse current upon illumination (Figure 3e), the HF‐SPD exhibits a marked photo‐dark current ratio (PDCR). More interestingly, dark current (*I*_dark_) and photo current (*I*_photo_) of HF‐SPD exhibit distinct dependences on the reverse bias. This apparently contradicts the classical *Φ*_B_‐lowering mechanism in conventional Ga_2_O_3_ photodetectors, wherein the constant thermal emission current under the reverse bias is raised by the photo‐lowered *Φ*_B_ but remains bias insensitive. Instead, the bias‐dependent reverse current in SBDs is normally ascribed to the interface state‐assisted tunnelling current. Therefore, *Φ*_B_ and interface states may be both optically tunable in In_2_O_3_ HF‐SPDs.

The responsivity (*R*), specific detectivity (*D* ^*^) and sensitivity of SPDs were calculated for quantitative comparison. *R* is expressed by the following relationship:^[^ ^28^ ^]^

(2) $$R=\frac{I_{photo}-I_{dark}}{P_{inc}S}$$

where *P*_inc_ and *S* represent the incident photo power density and the Schottky contact area, respectively. *D* ^*^ calibrates the anti‐noise capability of the photodetector and provides a normalized comparison benchmark for different detector devices. The following equation can be employed to obtain D^*^:^[^ ^10^ ^]^

(3) $$D^{*}=\frac{R\sqrt{S}}{\sqrt{2qI_{dark}}}$$

where *q* represents the electronic charge. Sensitivity can evaluate the weak photo detection capability of a detector, which can be characterized by the relationship defined below:^[^ ^6^ ^]^

(4) $$Sensitivity=\frac{I_{photo}-I_{dark}}{I_{dark}}$$

The calculation results of In_2_O_3_ SPD and HF‐SPD are presented in Figure 3g. The heterointerface architecture elevates the detection sensitivity by 80 times, revealing great potential of LT‐OSs in wide‐dynamic‐detection‐range photodetection. The sophisticated optimization of spatial defect distribution effectively overcomes the fundamental contradiction between defect‐boosted *I*_dark_ and state‐assisted *I*_photo_, enabling the excellent photo‐detection capability of LT‐OS HF‐SPDs.

Transient characteristics were further measured to assess the photoelectric dynamics. When In_2_O_3_ SPD and HF‐SPD were constantly biased at −2 V, 409‐nm‐*λ* illumination was applied at a photo power density of 1.5 mW cm^−2^ for 12 s. As demonstrated in Figure 2h, the HF‐SPD exhibits noticeably lower *I*_dark_ and higher sensitivity. Distinct from incumbent Ga_2_O_3_ SPDs, the In_2_O_3_ SPDs exhibit a persistent photoconductivity (PPC) effect.^[^ ^42^ ^]^ Similar PPC in LT‐OS phototransistors are normally ascribed to the gradual photoionization and slow recombination of V_o_, besides the instant excitation of photo carriers.^[^ ^43^ ^]^

The photoresponse time is calculated by fitting the dynamic photoelectric curve using the following double‐exponential relaxation equation:^[^ ^44^ ^]^

(5) $$J=J_{0}-Ae^{-\frac{t-t_{0}}{\tau_{1}}}-Be^{-\frac{t-t_{0}}{\tau_{2}}}$$

where *J*_0_ and *t*_0_ are steady‐state *I*_photo_ density and light‐on time, respectively, *A* and *B* are two constants, and *τ*_1_ and *τ*_2_ represent the response times of relaxation processes. As indicated in Figure S12 (Supporting Information), the response time of In_2_O_3_ SPD is 686.0 ms, while HF‐SPD exhibits a faster response time of 367.7 ms. Despite the alike curves, the quality of the Schottky interface has a determinative influence on the underlying photoelectric dynamics.

It is noteworthy that the low *I*_dark_ of HF‐SPD also inspires the potential for weak light detection, especially considering that *I*_photo_ exponentially increases with reverse bias. When the power density of 409‐nm‐*λ* light is reduced to only 1 µW cm^−2^, the In_2_O_3_ SPD did not respond at all, while the HF‐SPD still exhibited observable photoresponse (Figure S13, Supporting Information). The comprehensive photodetection performance metrics of In_2_O_3_ SPD and HF‐SPD are presented in Figure S14 (Supporting Information), clearly demonstrating the superior photodetection functionality of LT‐OS HF‐SPD.

### 2.4 Comprehensive Analysis on Photoelectric Properties of In_2_O_3_ HF‐SPD

**Figure 4:**
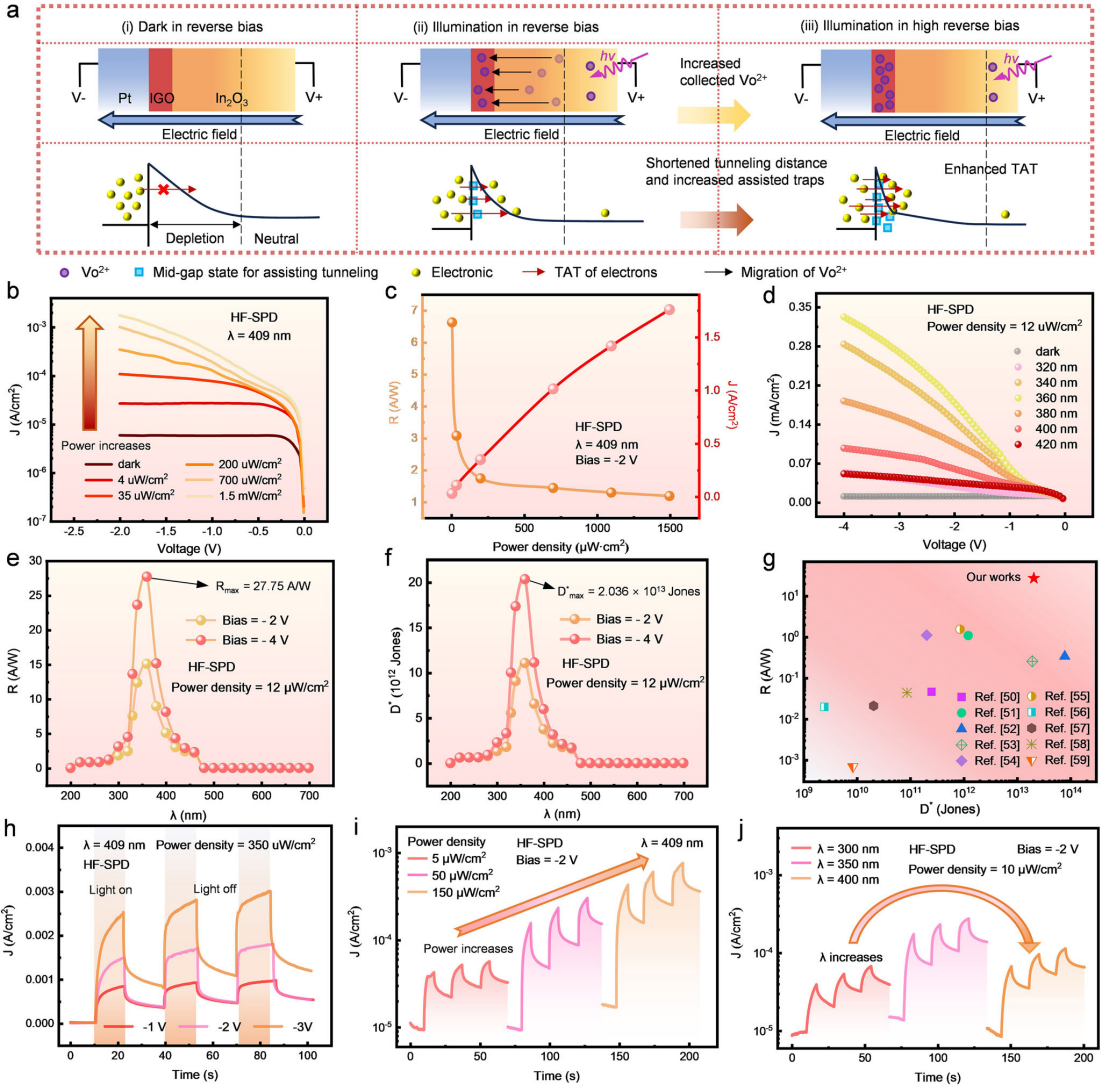
Photoresponse mechanism and performance metrics of In_2_O_3_ HF‐SPD. a) The photodetection mechanism of In_2_O_3_ HF‐SPD. b) *I–V* characteristics of HF‐SPD subjected to 409‐nm illumination at various optical power densities. c) The power density dependences of *R* and *J* extracted at a reverse bias of −2 V. d) *I–V* characteristic curves of HF‐SPD subjected to 12 µW cm^−2^ illumination with various wavelengths. e) *R*‐*λ* and (f) *D* ^*^‐*λ* curves extracted at reverse biases of −2 and −4 V. g) Benchmark of the state‐of‐the‐art demonstrations of OS photodetectors. Dynamic photoresponse of HF‐SPD under h) various reverse biases, i) photo power densities, and j) wavelengths, respectively.

As mentioned in Section 2.3, the photoelectric properties of In_2_O_3_ HF‐SPD are found to root in the photoionization and migration dynamics of Vo. The underlying mechanisms and corresponding simulations are shown in **Figures** **4**a and S15 (Supporting Information). (i) In the reverse‐biased SPD, the depletion region is widened while *Φ*_B_ stays high, resulting in a low and bias‐independent *I*_dark_ (Figure 4b). This is consistent with the thermal emission (TE) mechanism,^[^ ^45^ ^]^ as confirmed by the well fitted *J*_dark_‐*V*_A_ curves (Figure S15d, Supporting Information). (ii) Under illumination, the neutral Vo states in In_2_O_3_ are optically ionized into positively charged Vo^2+^ and electrons (Figure S15b, Supporting Information). The low electron concentration in the depletion region leads to low recombination rate and thus long life‐time of Vo^2+^ defects, which accumulates in the IGO interface layer (Figure S15c, Supporting Information) under the electric field extraction effect (EFEE),^[^ ^44^ ^]^ as declared in Note S3 (Supporting Information). EFEE effectively separates photogenerated electrons and Vo^2+^, thereby reducing their recombination rate and increasing their lifetime. These interface Vo^2+^ states assist more abundant electrons to tunnel from anode into OS through the ultrathin HfO_2_,^[^ ^34^, ^46^ ^]^ as illustrated in Note S4 (Supporting Information). Thus, the low‐Vo IGO interface layer is endowed with a functional auxiliary photo detection capacity. (iii) As the bias voltage increases, a greater amount of Vo^2+^ accumulates near the metal side of the depleted ultrathin IGO. The Vo^2+^ at the interface possesses an energy level distribution, and a greater bias voltage can activate more abundant TAT from each energy level. As a result, the probability of TAT increases significantly, and *I*_photo_ can exhibit a substantial or even exponential increase as the reverse‐biased voltage increases. The well‐fitted *J*_photo_‐*V*_A_ curves verify the combined effects of EFEE and TAT mechanisms (Figure S15d, Supporting Information). Notebly, the narrowed triangular band diagram of the interface depletion region further strengthens the TAT mechanism of deep‐level trap states, contributing to the noticeably high photo‐response to short‐wavelength illumination. In summary, the LT‐OS HF‐SPD realizes ultra‐low dark‐state current and functionalizes superior photo response through the synergistic regulation of trap state distribution and photoelectron transportation. Such internal tangling of photo response and electric field endows LT OSs with enormous photodetection potentials in complex environments.

In order to further explore the photodetection functionalities of In_2_O_3_ HF‐SPD, varied optical power densities and wavelengths were implemented. As the optical power density rises from 4 µW cm^−2^ to 1.5 mW cm^−2^, *I*_photo_ increases notably and its bias dependence becomes more pronounced (Figure 4b). Most plausibly, more photoexcited Vo^2+^ states accumulate at the Schottky interface and thus considerably enhance the TAT current. The dependences of *I*_photo_ and *R* on illumination power density (Figure 4c) demonstrate consistently high photoresponse across the wide optical power range.

As a pivotal performance indicator of photodetector in converting incident photons into effective electrical signals, EQE is calculated according to the following equation:^[^ ^47^ ^]^

(6) $$\mathrm{EQE}=\frac{\left(I_{photo}-I_{dark}\right)/q}{P_{inc}/h\nu }$$

where *h* and *ν* respectively represent the Planck constant and the frequency of photons in a vacuum.

The dependences of *D* ^*^ and EQE on photo power density are also extracted and presented in Figure S16 (Supporting Information). With the increasing illumination power density, abundant photogenerated carriers in the space charge region may enhance the recombination of photoionized Vo, thus reducing the fraction of Vo^2+^ population which can reach the Schottky interface and constraining the boosting effectiveness of *I*_photo_ with the illumination power. Accordingly, In_2_O_3_ HF‐SPD exhibits much higher *D* ^*^ and EQE under weak illumination, perfect for the high‐dynamic photodetection.

The proposed HF‐SPD architecture also endows wide‐bandgap OSs with the specific‐spectrum detection capability. As shown in Figure 4d, HF‐SPDs exhibit pronounced photoelectric responses to the UVA spectrum (320–420 nm). The wavelength response characteristics are also evaluated using the *R*‐*λ*, *D* ^*^‐*λ*, and EQE‐*λ* curves, respectively presented in Figures 4e,f, and S17 (Supporting Information). The In_2_O_3_ HF‐SPD not only possesses noticeable photoresponse to UVA spectrum, but more importantly, exhibits a sensitive wavelength selectivity. This paves the way of LT‐OS SPDs to the intelligent photodetection applications.

In Figure 4e, *I*_photo_ of In_2_O_3_ HF‐SPD appears as *λ* decreases to shorter than 480 nm. This might be attributed to the corresponding photon energy gradually exceeding the energy level of the Vo defects, which are located at the valence band maximum (VBM) and approximately extends to the range of 1 eV above VBM.^[^ ^48^ ^]^ As *λ* further decreases into UVB spectra (280–320 nm), the photoresponse is gradually mitigated, distinct from traditional Ga_2_O_3_ PDs.^[^ ^49^ ^]^ As illustrated in Figure S18 (Supporting Information), the short‐*λ* illumination activates the electron transition from the valence band (*E_v_*) to *E_c_*. Nevertheless, the abundant trap states may effectively strengthen the combination between excited electrons and photoionized Vo, thus weakening the photocurrent gain. Additionally, excessive photon energy may also lead to severe non‐radiative recombination, which also reduces the photoresponse.

In summary, only photons of proper energies can optimize the photoexcitation‐recombination trade‐off to enable superior photodetection capability. The most excellent performance metrics are achieved at the wavelength of 360 nm, including *R*_max_ of 27.75 A W^−1^ and *D* ^*^_max_ of 2.036 × 10^13^ Jones. Figure 4g and Table S1 (Supporting Information) clearly exhibit the comprehensive benchmark comparison between HF‐SPD and previously reported OS photodetectors.^[^ ^50^, ^51^, ^52^, ^53^, ^54^, ^55^, ^56^, ^57^, ^58^, ^59^ ^]^ The heterointerface architecture and electrical bias synergistically regulate the distribution and transportation of mid‐gap states and photoelectrons, thereby enabling superior unique selective UVA detection capabilities.

The dynamic photoelectric characteristics of HF‐SPDs are further investigated. The time evolutions of *I*_photo_ follow the same dependences on bias voltage, illumination wavelength and power density, as shown in Figure 4h–j. In the reverse‐biased In_2_O_3_ HF‐SPD, photoionized Vo (Vo^2^⁺) defects are separated from electrons and then gradually accumulated into the depleted IGO interlayer, continuously enhancing the TAT *I*_photo_, until a generation‐recombination equilibrium between Vo^2^⁺ and electron is finally achieved, corresponding to the gradually saturated *I*_photo_. Such photoelectric dynamics may be accelerated through device architectures and bias modulation, as previously demonstrated in LT‐OS phototransistors.^[^ ^60^ ^]^

When the light is turn off, *I*_photo_ exhibits an instantly drop but stays high for a quite long period. This is almost identical to the persistent photoconductive effect (PPC) in reported LT‐OS phototransistors,^[^ ^61^ ^]^ deriving from long‐lifetime Vo^2^⁺ defects in the low‐electron‐concentration depleted OS regions near Schottky interface or gate insulator interface. Such PPC effect has been successfully eliminated in OS phototransistors by proposing a positive *V*_g_ pulse to induce abundant electrons to accelerate the recombination of Vo^2^⁺. As demonstrated in Figure S19 (Supporting Information), a forward bias pulse has realized the same PPC‐elimination efficiency in HF‐SPD, since electrons abundantly emit over the bias‐lowered Schottky barrier into OS and instantly recombinant with Vo^2^⁺ (Figure S20, Supporting Information). This further confirms that the Vo^2^⁺ population at Schottky interface directly dominates the photoresponse of HF‐SPD.

### 2.5 Intelligent Fire Detection Based on HF‐SPD

**Figure 5:**
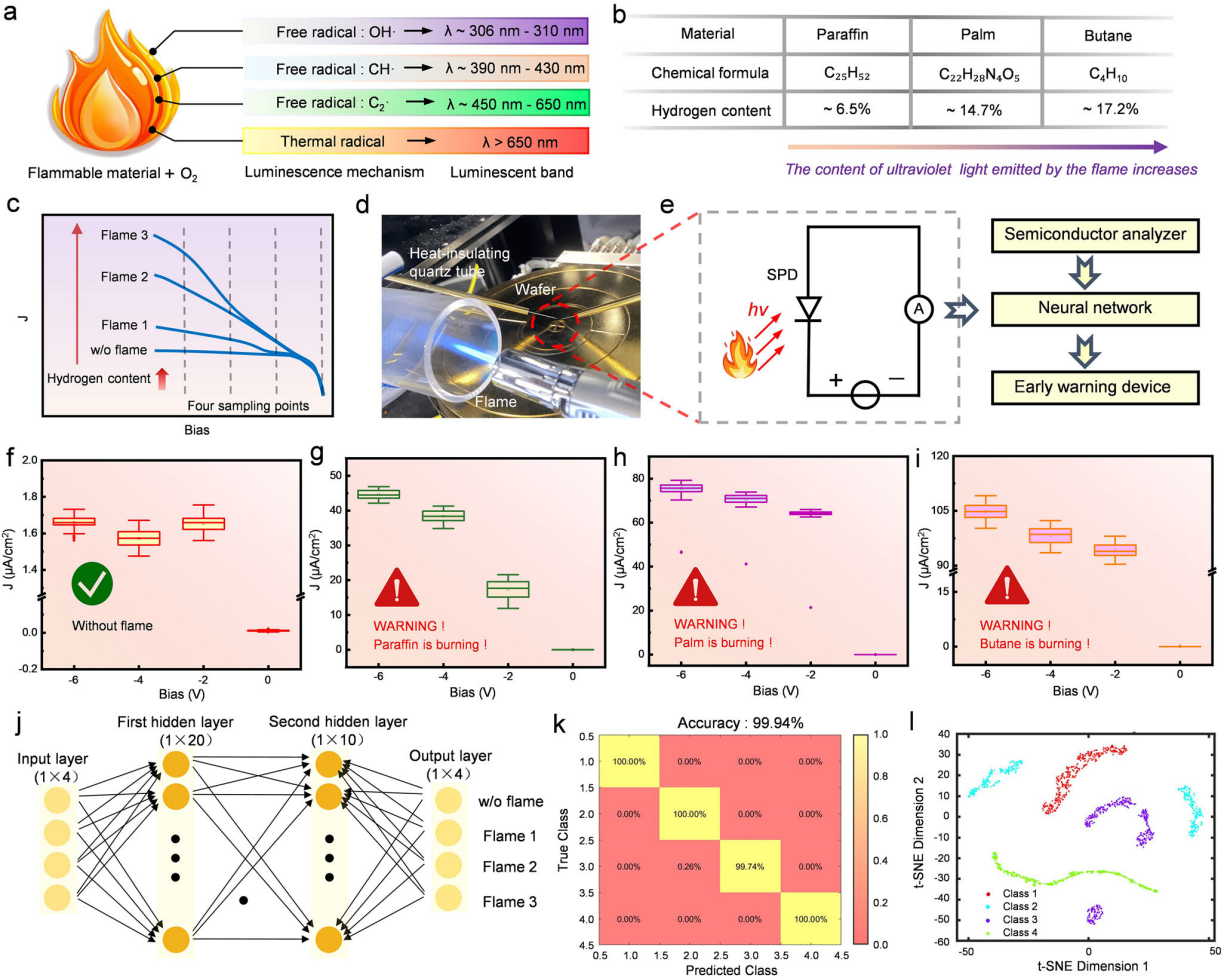
Intelligent fire detection based on HF‐SPD and MLP algorithm. a) The correspondence between the free radicals from combustions and the wavelengths of flame light. b) The chemical formulas and hydrogen content of different combustion materials. c) I‐V curves of HF‐SPDs subjected to different flame lights, with the sampling bias points illustrated. d) Photograph of the flame detection test. e) Technical maps of an intelligent fire detection application. f–i) Data sets of conditions without flames, paraffin burning, palm burning, and butane burning, respectively. j) Structural schematic diagram of the MLP neural network algorithm. k) Confusion matrixes of the HF‐SPD for identifying flames. l) The t‐SNE visualization results of the HF‐SPD for identifying flames.

The fire accident is one of the most common natural disasters, which pose serious threats to human life and property safety. During the incipient stage of a fire, the prompt detection of weak flames and the accurate identification of combustible materials are essential for delivering early fire alerts and facilitating effective fire suppression.^[^ ^8^, ^62^ ^]^ Based on the multidimensional and nonlinear photoresponse of LT‐OS HF‐SPD, the multi‐layer perceptron (MLP) neural network was incorporated to develop an intelligent fire early warning system for the sensitive detection and precise classification. As illustrated in **Figure** **5**a, the free radicals of CH· groups during the combustion process illuminate the UVA light (390–430 nm), which can be precisely detected by the In_2_O_3_ HF‐SPD.^[^ ^6^ ^]^ Higher hydrogen content of the combustion material results in higher UV intensity of the flame. Figure 5b shows the chemical formulas and corresponding hydrogen contents of three common combustion materials (paraffin, palm, and butane). As shown in Figure S21 (Supporting Information), their combustions emitted different color glow and the UV intensities were quantitatively calibrated at 320 nm to be 75.14, 89.26, and 112.96 µW cm^−2^, consistent with their relative hydrogen contents.

As demonstrated in Figure 5c, the wavelength‐selective photoresponse of the HF‐SPD enables the capability to distinguish the flame lights from different combustion materials. The unique bias‐dependent *I*_photo_ is utilized to conduct multi‐feature point sampling of flame light, ensuring the accuracy of both detection and recognition. The HF‐SPD was successively biased at 0, −2, −4, and −6 V for high‐frequency detecting of flame light. Multi‐channel bias sampling enables the accurate real‐time extraction of flame light features, providing a critical foundation for the intelligent identification of combustion materials. The HF‐SPD exhibits a response time on the order of hundreds of milliseconds (Figure S12, Supporting Information), which is faster than second‐scale commercial fire detectors and thus ensures the instantaneous detection of the weak flame in the early stage of fire. It should be noted that the flame was enveloped by an insulated and transparent quartz tube, which shielded the device from the effects of heating during tests (Figure 5d). Figure 5e illustrates the technical process for the early warning and classification of burning materials. The semiconductor analyzer was employed for the real‐time and high‐frequency photocurrent sampling, and then the measured data set was calculated and classified using an MLP neural network. Two thousand data points were sampled at each bias voltage to ensure the accuracy of the calculation. Figure 5f–i demonstrates the current density datasets of the HF‐SPD in conditions of a normal lighting environment without flame combustion and different materials burning. Attributed to the excellent uniformity and stability of the HF‐SPD, the datasets measured under similar environmental conditions exhibit the concentrated distribution trend, which provides reliable data support for the detection of flames and the accurate classification of combustion materials.

Figure 5j is the structural diagram of the MLP neural network. The number of neurons in the input layer of the neural network should be consistent with the number of sampled features. Consequently, the input layer consists of only four neurons. This model contains two hidden layers, consisting of 20 and 10 neurons respectively, which extract the high‐order features of the data through a nonlinear activation function. The Softmax function was employed to output the probabilities of each category in the final classification task. The first 80% of the collected dataset was utilized for the computational training of the neural network, while the remaining 20% was reserved for testing. As the training progressed, the cross‐entropy decreased significantly, which is one of the crucial loss functions in neural network computing. As exhibited in Figure S22 (Supporting Information), the best validation performance was achieved after 134 training sessions. As shown in Figure 5k, the confusion matrix reveals that the HF‐SPD exhibits a high classification accuracy rate of 99.94% for various combustion materials. This exceeds the prescribed accuracy for fire alarm systems and provides the vital fire information for targeted fighting responses. The samples of the same category were grouped, and the t‐distributed stochastic neighbor embedding (t‐SNE) was utilized to visually assess the concentration of the dataset. The high‐dimensional data features output from the fully connected layer were mapped to the 2D space, and the data samples formed distinct clusters, as shown in Figure 5l.

The wavelength‐selective HF‐SPD effectively extracts the broadband optical information of different flames, and the bias‐tunable photoresponse further ensures multidimensional datasets for accurate classification of combustion materials. Moreover, the powerful scalability of HF‐SPD device and neural network algorithm unlocks the inspiring prospects for intelligent early warning systems targeting multi‐component combustion scenarios. The low‐temperature fabrication advantage of LT‐OS HF‐SPDs paves the way for ubiquitous optoelectronic applications, including flexible photodetection and ICS systems.

## 3 Conclusion 

A novel heterointerface strategy was proposed to functionalize the promising photoelectric properties of LT‐OSs in Schottky photodiodes. By sophisticatedly tailoring the spatial distribution of mid‐gap states at the Schottky interface and within the OS bulk, the interface defect‐induced Fermi level pinning is considerably mitigated and thus enables an ultralow dark current, while the photoionization of bulk Vo and field‐assisted tunnelling of electrons further realize the distinctive multidimensional photoresponse with wavelength selectivity and bias tunability. Within the UVA band, a high responsivity of 27.75 A W^−1^ and a superior specific detectivity of 2.036 × 10^13^ Jones were demonstrated. With the assistance of MLP neural network algorithms, the multidimensional photoelectric data extracted from HF‐SPD enables the precise pattern recognition of the flame spectrum. Such an intelligent fire warning system accurately classifies the flame spectra of various burning materials with a noticeably high accuracy rate of 99.94%. This innovative LT‐OS HF‐SPD holds significant promise for low‐temperature PDs in ubiquitous optoelectronic applications, such as flexibility‐enabled wearable PDs and heterointegration‐compatible ISC systems.

## 4 Experimental Section

### Device Fabrication

The commercial Corning glass substrate was ultrasonically cleaned in dehydrated ionized water for 20 min. 40 nm In_2_O_3_ and 8 nm IGO were successively deposited on the substrate surface by magnetron sputtering. During the deposition process, the chamber pressure was maintained at 0.36 Pa, and the flux ratio of the introduced Ar: O_2_ was adjusted to 10:24 sccm, followed by photolithography patterning. The active layer was etched using HCl/H_2_O with a volume fraction ratio of 1:1. After removing the photoresist with acetone and alcohol, N_2_O plasma treatment and annealing at 400 °C were performed successively.

PECVD was used for the deposition of the SiO_2_ passivation layer. The gas pressure during the reaction was 0.7 Torr, and the gas flow rates of SiH_4_ and N_2_O were 100 and 710 sccm, respectively. The passivation layer was patterned employing RIE. HfO_2_ with a thickness of 2 nm was deposited by ALD as the dielectric insertion layer. The deposition temperature was 150 °C, and the precursor was TEMAHf. Then, N_2_O plasma treatment and annealing at 400 °C were performed again to repair the impact damage of the plasma on the active layer. Sequentially, Pt/ITO (20 nm/50 nm) and ITO (70 nm) were respectively deposited by magnetron sputtering as the anode and cathode metals of the SPD. Each SPD photodetector unit was an interdigital asymmetrical structure with 10 pairs of fingers. The length, width, and spacing were 240, 10, and 30 µm, respectively. Annealing at 300 °C was used for oxygen supplementation and enhanced conductivity of ITO transparent electrodes.

### Device Characterization

Scanning Electron Microscopy (SEM, Quanta 650), Field Emission Transmission Electron Microscope (TEM, JEM‐2100F), and Energy Dispersive Spectroscopy (EDS, JEM‐2100F) were utilized to identify microscopic morphology and elemental composition. UV–vis spectrophotometer (Evolution 350) characterized the optical transmission spectra of thin films. X‐ray Photoemission Spectroscopy (XPS, Nexsa) studied the oxygen vacancy concentration of the film, and Ultraviolet Photoemission Spectroscopy (UPS, Nexsa) was used to calibrate the work function of the materials and energy level of *E_v_*.

The incident monochromatic light (409 nm) power was calibrated by a Si photodetector (LE‐LPM‐10AP). Xenon lamp sources (LSH‐X500) and spectrometers (Zolix omni‐λ300) were used to apply illumination excitation with different wavelengths. The intensities of illumination and fire flame were calibrated using a standard optical power meter (TOHRLABS‐PM100D). The semiconductor parameter analyzer (Agilent B1500) was utilized to evaluate the photoelectric performance.

### Date Extraction of Fire Flame

The photocurrent of HF‐SPD under four biases (0, −2, −4, −6 V) were sampled at 100 Hz using the semiconductor parameter analyzer (Agilent B1500).

## Conflict Interest

The authors declare no conflict of interest.

## Supporting information



Supporting Information

## Data Availability

The data that support the findings of this study are available from the corresponding author upon reasonable request.
