# Supplementary material for: Heterointerface‐Functionalized Photoelectric Response of Metal‐Oxide Schottky Photodiode for Intelligent Fire Detection
Source: Adv Sci (Weinh). 2025 Dec 3;13(9):e19318. doi: 10.1002/advs.202519318 (PMC12903981; doi:10.1002/advs.202519318)
Supplement: Supplementary file 1 — Supporting Information [file ADVS-13-e19318-s001.docx]

**Supporting information**

**Heterointerface-Functionalized Photoelectric Response of Metal-Oxide Schottky Photodiode for Intelligent Fire Detection**

*Yuyang Cai, Zhiwei Zheng, Zhiwu Zhong*, *Yuhan Zhang, Tengyan Huang, Yucheng Cao, Dawei Zheng, Yen Hung Lin, Fion Sze Yan Yeung, Kuan-Chang Chang, Jie Chen, Hoi Sing Kwok, Lei LU^*^, Yufeng Jin^*^*

Yuyang Cai, Zhiwei Zheng, Zhiwu Zhong, Yuhan Zhang, Tengyan Huang, Yucheng Cao, Kuan-Chang Chang, Jie Chen, Lei LU, Yufeng Jin

School of Electronic and Computer Engineering, Peking University, Shenzhen 518055, China.

Email: lulei@pku.edu.cn, yfjin@pku.edu.cn

Dawei Zheng, Yen Hung Lin, Fion Sze Yan Yeung, Hoi Sing Kwok

State Key Laboratory of Displays and Opto-Electronics, The Hong Kong University of Science and Technology, Clear Water Bay, Kowloon, Hong Kong SAR.

Keywords: Schottky photodiode, oxide semiconductor, low temperature, heterointerface, intelligent photodetection application

**Supplementary Notes**

**Note S1: The ultrathin dielectric layer achieving Fermi level de-binding**

MIGS is one of the fundamental mechanisms that determines the height of the Schottky barrier.^[1]^ The conduction bands of the metal and semiconductor exhibit a partial energetic overlap within the bandgap region in the contact interface. The metal electron wave function penetrates into the semiconductor bandgap, introducing additional gap states. For the energy within the band gap, the wave vector obtained by solving the Schrodinger equation is complex, which decays exponentially in space. Its imaginary component describes the exponential decay characteristic of the wave function. A larger imaginary part results in a faster attenuation of the wave function with distance, leading to a smaller penetration depth (δ) of MIGS.

The electron wave function of metals decays more rapidly and exhibits a shallower penetration depth in wide bandgap materials. The insertion of a wide-bandgap interlayer between a metal and a semiconductor can effectively attenuate the metal electron wave function and reduce the charge transfer, thereby suppressing its penetration into the semiconductor interface. HfO_2_ is a commonly used high-k gate dielectric in very large-scale integrated circuit devices, which possesses a wide bandgap of approximately 6 eV. In addition, the *E_c_* of HfO_2_ is lower than that of other wide bandgap insulating layers, which offers lower forward conduction resistance for SPD.

**Note S2: The Cheung method for extracting the basic parameters of Schottky diodes**

The traditional thermal-electron emission model is utilized to analyze the current-voltage (*J-V*) relationship of Schottky diodes:^[2]^

| $J=J_{0}\left[ exp\left( \frac{q(V-JR_{s}}{nkT} \right)-1 \right]$ | (1) |
| --- | --- |
| $J_{0}=A^{*}T^{2}\left[ exp\left( \frac{-q\Phi_{B}}{kT} \right) \right]$ | (2) |

where the *A^*^* represents the effective Richardson constant of amorphous oxide semiconductor, which is approximately 41 A·cm^−2^·K^−2^. The *k* is the Boltzmann constant. The *R_S_* and *Φ_B_* represent the series resistance and the Schottky barrier height, respectively. The n is the ideal factor. When a diode is subjected to a bias voltage *V= V_D_+IR_S_*, the following relationship can be derived from Equation (1):

| $V=R_{s}A_{eff}J+n\Phi_{B}+\frac{nkT}{q}ln\left( \frac{J}{A^{*}T^{2}} \right)$ | (3) |
| --- | --- |

where *A_eff_* is the effective area of Schottky contact. Differentiating *V* with respect to *ln(J)* yields the following formula:

| $\frac{dV}{d(lnJ)}=R_{s}A_{eff}J+\frac{nkT}{q}$ | (4) |
| --- | --- |

Draw the line from *dV/d(lnJ)* to *J*, and the ideal factor n can be obtained based on the intercept of this line to the Y-axis. The *H(J)* is introduced and its expression can be described as:

| $H\left( J \right)=V-\frac{nkT}{q}ln\left( \frac{J}{A^{*}T^{2}} \right)$ | (5) |
| --- | --- |

Substituting Formula (5) into Formula (3), the following relationship can be obtained:

| $H\left( J \right)=R_{s}A_{eff}J+ n\Phi_{B}$ | (6) |
| --- | --- |

Draw the graph of *H(J)~J*, and the Schottky barrier *Φ_B_* can be obtained based on the intercept of this line to the Y-axis.

**Note S3: Electric field extraction effect (EFEE) in OS**

EFEE is a vital carrier transmission mechanism in FET based on OS. The physical essence of EFEE lies in the directional modulation of the motion of ionized oxygen vacancies (Vo²⁺) and electrons by an external electric field. As the primary charge carriers in oxide semiconductors (OS), their kinetic behavior is strongly dependent on the gate bias conditions. By applying specific dual-gate voltage sequences, the vertical electric field distribution within the channel can be precisely controlled. This enables spatial reorganization of carrier concentration in the channel, leading to a significant enhancement of the device's photo-response speed.^[3]^

In two-terminal devices for photo detection, EFEE modulates the electric field strength within the depletion region, facilitating effective separation of photo-generated Vo²⁺ and electrons. This process not only prolongs the lifetime of trap states but also causes these states to accumulate at the Schottky interface, thereby providing an efficient assisted-tunneling pathway for carriers. At the microscopic level, the recombination of interface states induced by EFEE, combined with band bending, synergistically enhances carrier injection and extraction efficiency. This mechanism serves as the fundamental physical basis for achieving high photoelectric gain in HF-SPDs**.**

**Note S4: The carrier conduction mechanism of the oxide SPD**

The carrier conduction mechanism in the oxide SPD primarily involves thermionic emission (TE) and trap-assisted tunneling (TAT).^[4]^ TE describes the process whereby electrons in the metal gain sufficient thermal energy to surmount the Schottky barrier at the metal–semiconductor interface, thereby contributing to current. The height of the Schottky barrier directly influences the strength of TE. Obviously, the inherent barrier of the metal-semiconductor contact determines that TE is very weak.

TAT is a carrier transport mechanism unique to oxide Schottky diodes, which is the most crucial source of photoelectric gain. The neutral trap energy levels in oxide semiconductors tend to capture free electrons. Under the reverse bias voltage, the captured carriers can effortlessly tunnel from the trap level to the semiconductor conduction band. The probability of TAT is closely related to the width of the barrier region and the intensity of the applied electric field. The trap state serves as the central hub for electron transport between metals and semiconductors. Thus, TAT dominates the carrier transport mechanism of SPD in the photoelectric detection mode.

**Supplementary Figures**


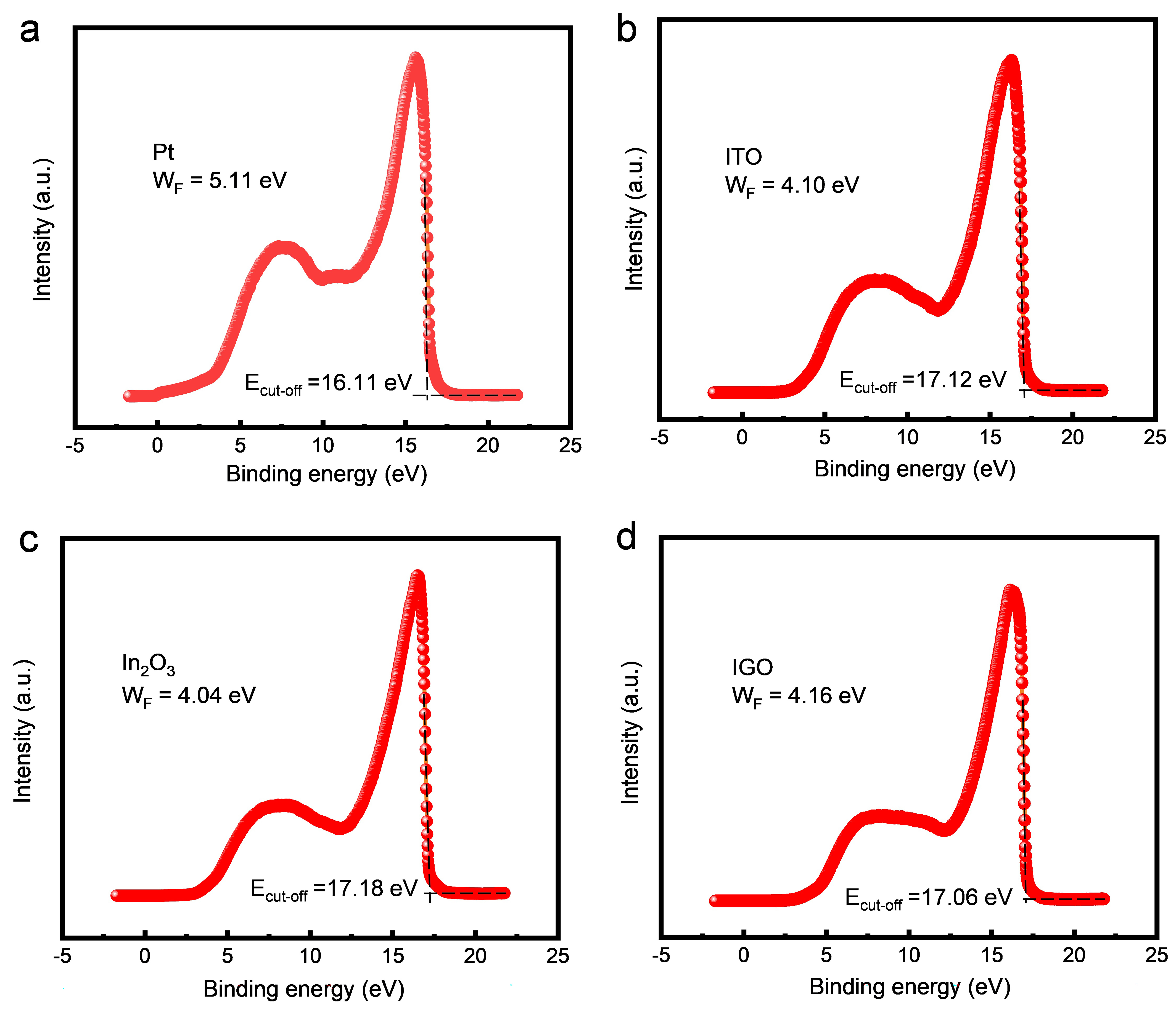


**Figure S1.** Work function of Pt, ITO, In_2_O_3_ and IGO, respectively**.**


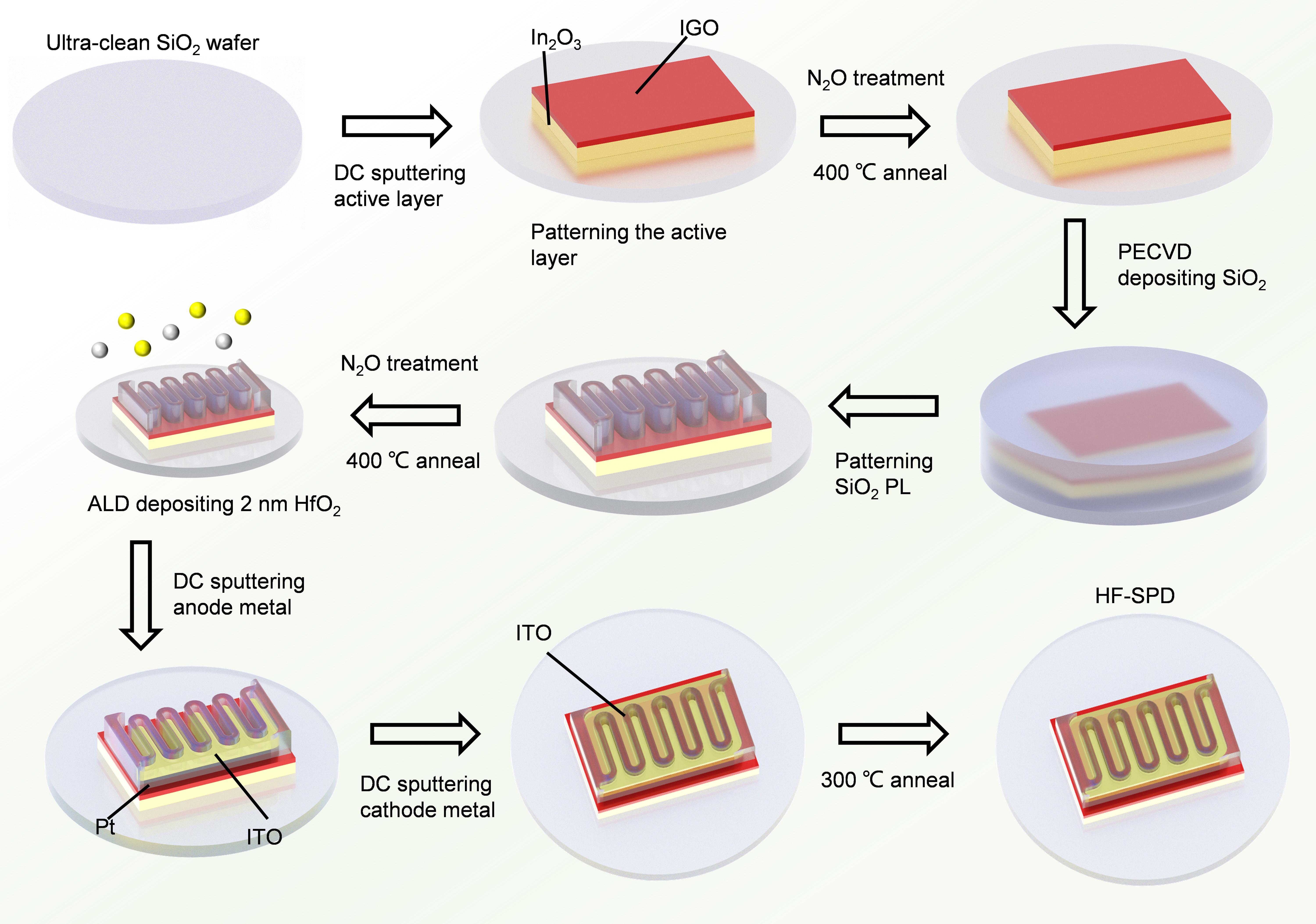


**Figure S2.** Overview diagram of the preparation process of the HF-SPD.


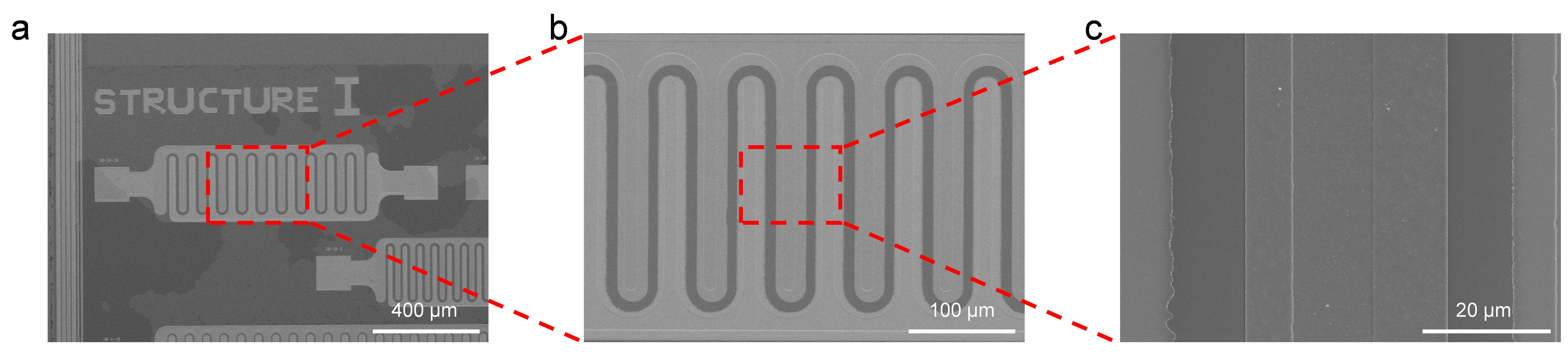


**Figure S3.** SEM images of the HF-SPD.


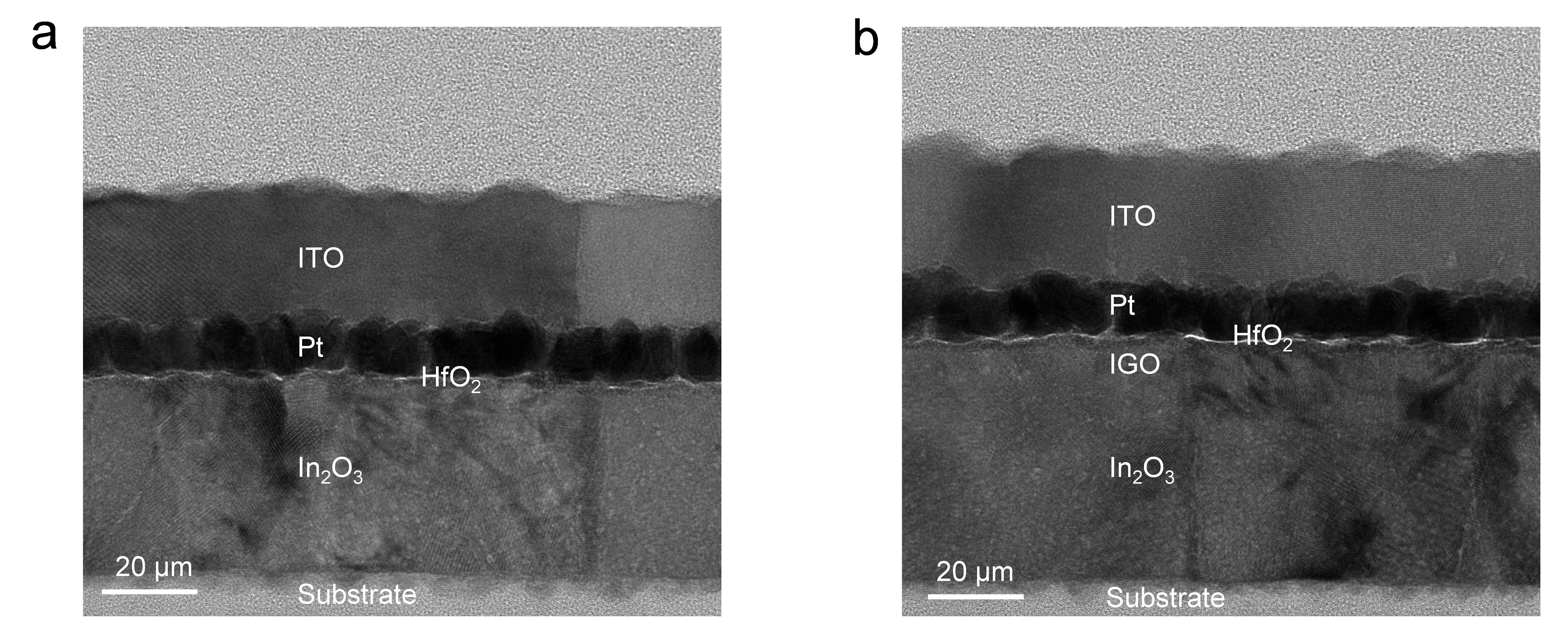


**Figure S4.** Cross-section TEM morphologies of (a) the In_2_O_3_ and (b) HF-SPD.


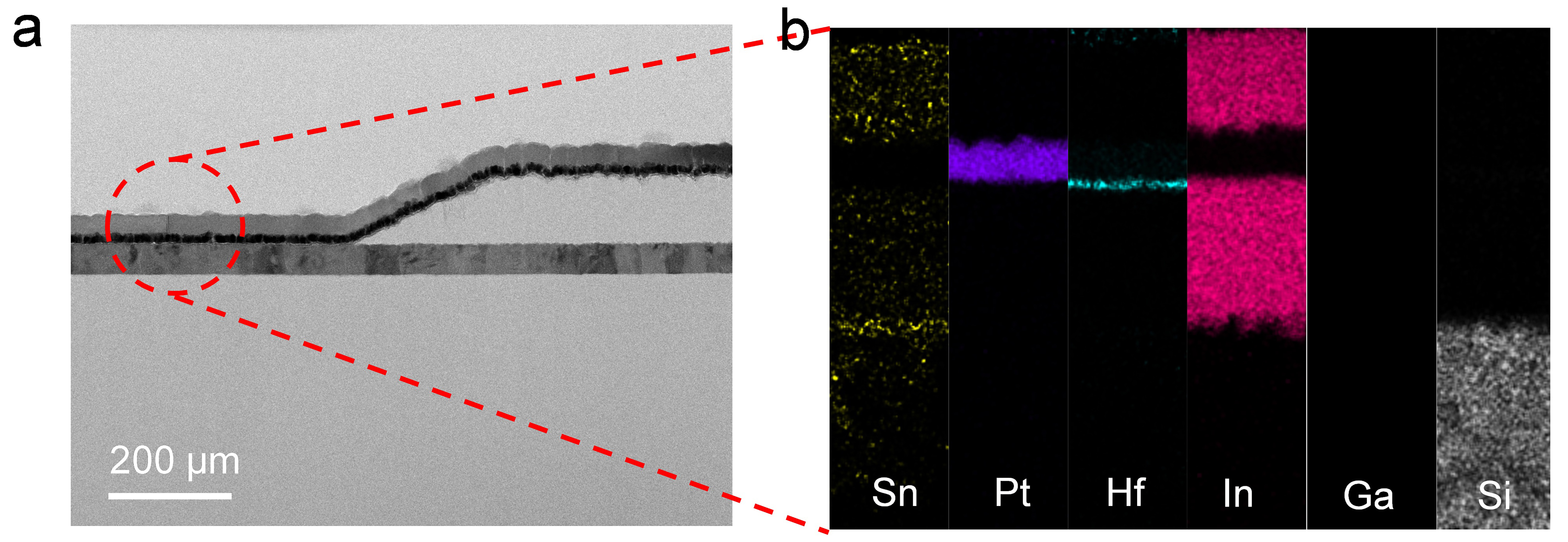


**Figure S5.** TEM morphology and EDS elemental component analysis of the In_2_O_3_ SPD.


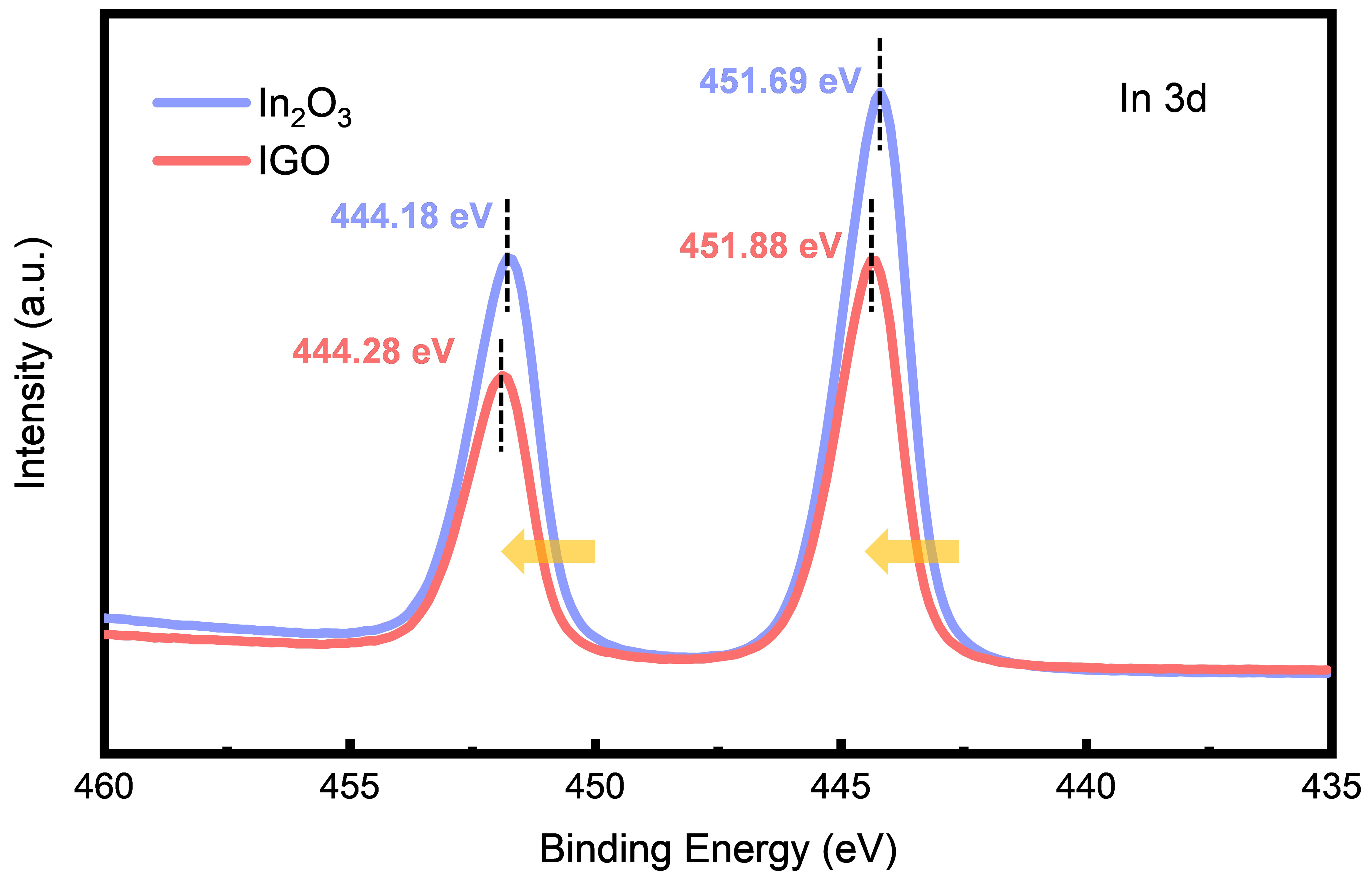


**Figure S6.** XPS In 3d spectra of In_2_O_3_ and IGO.


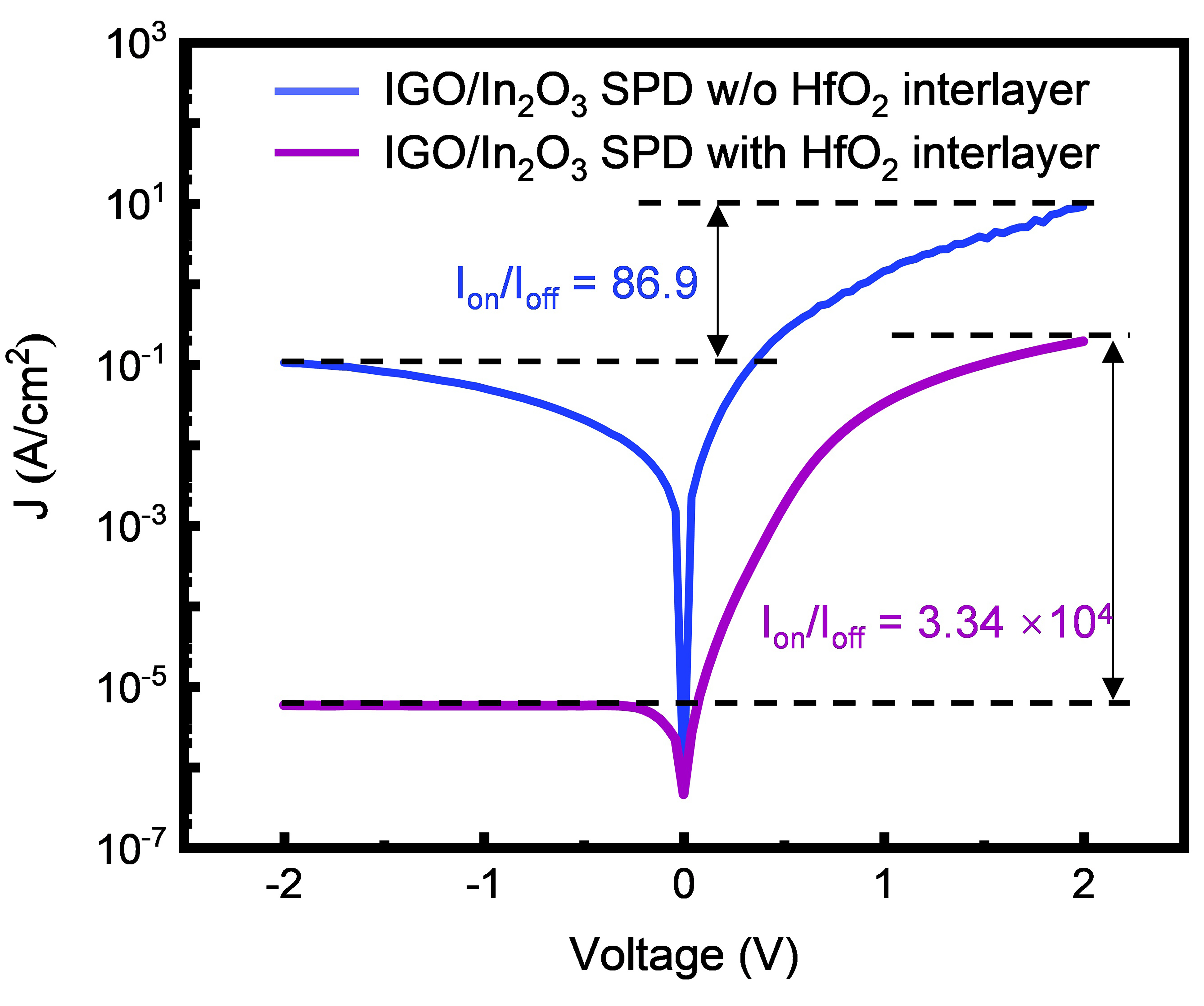


**Figure S7.** *J*-*V* characteristics of In_2_O_3_ SPD and IGO/In_2_O_3_ SPD without and with the HfO_2_ interlayer**.**


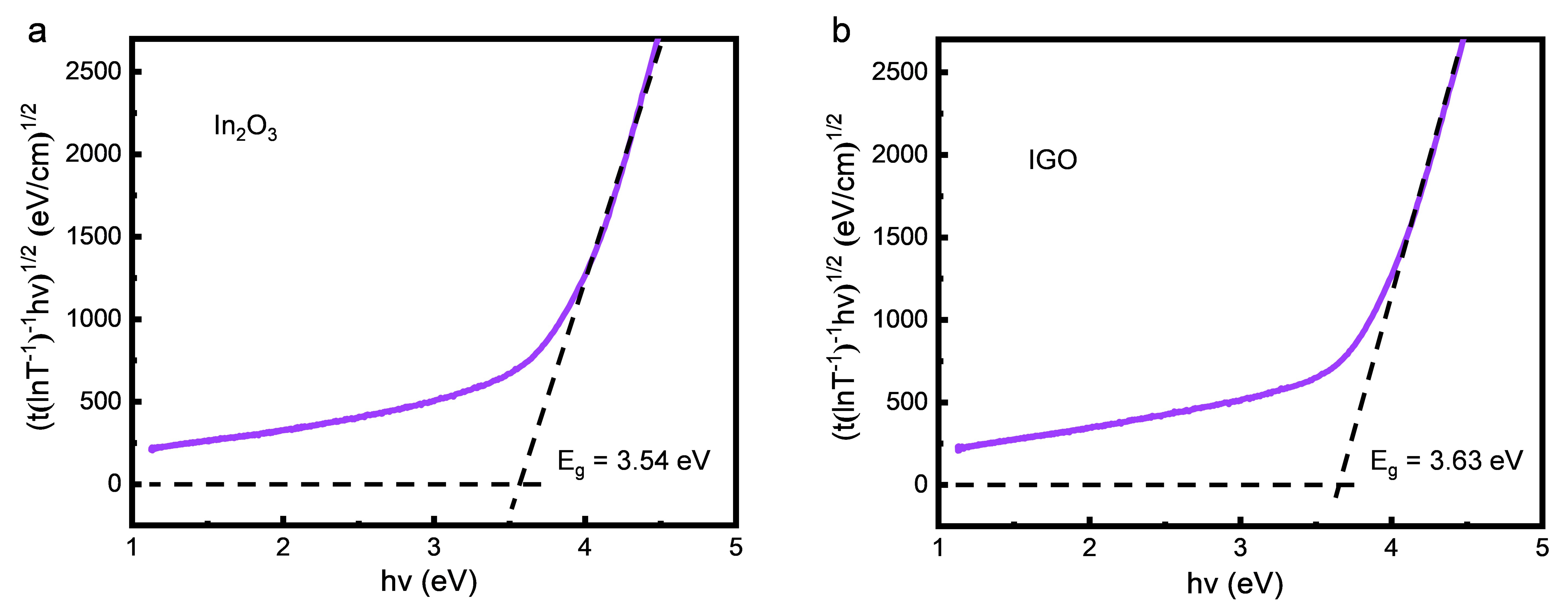


**Figure S8.** *E_g_* of In_2_O_3_ and IGO extracted by the Tauc plotting method.


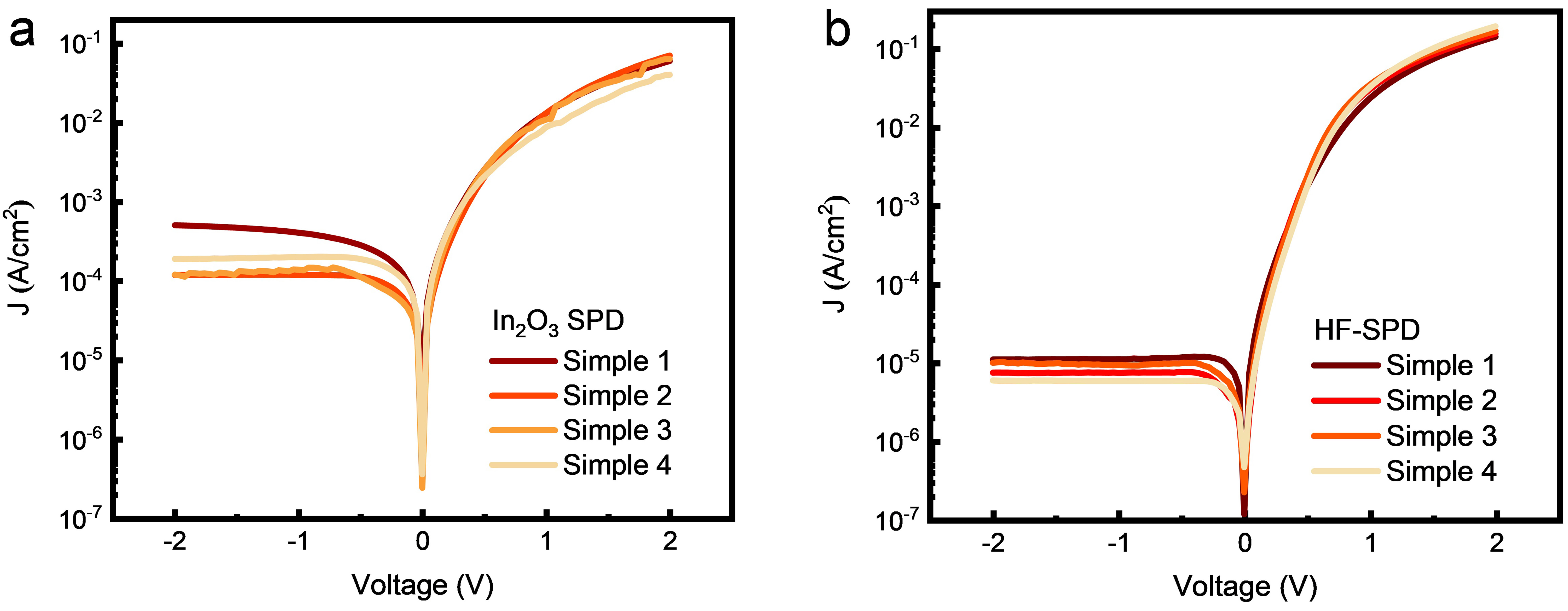


**Figure S9.** Uniformity comparison of rectification characteristics of the In_2_O_3_ SPD and HF-SPD.


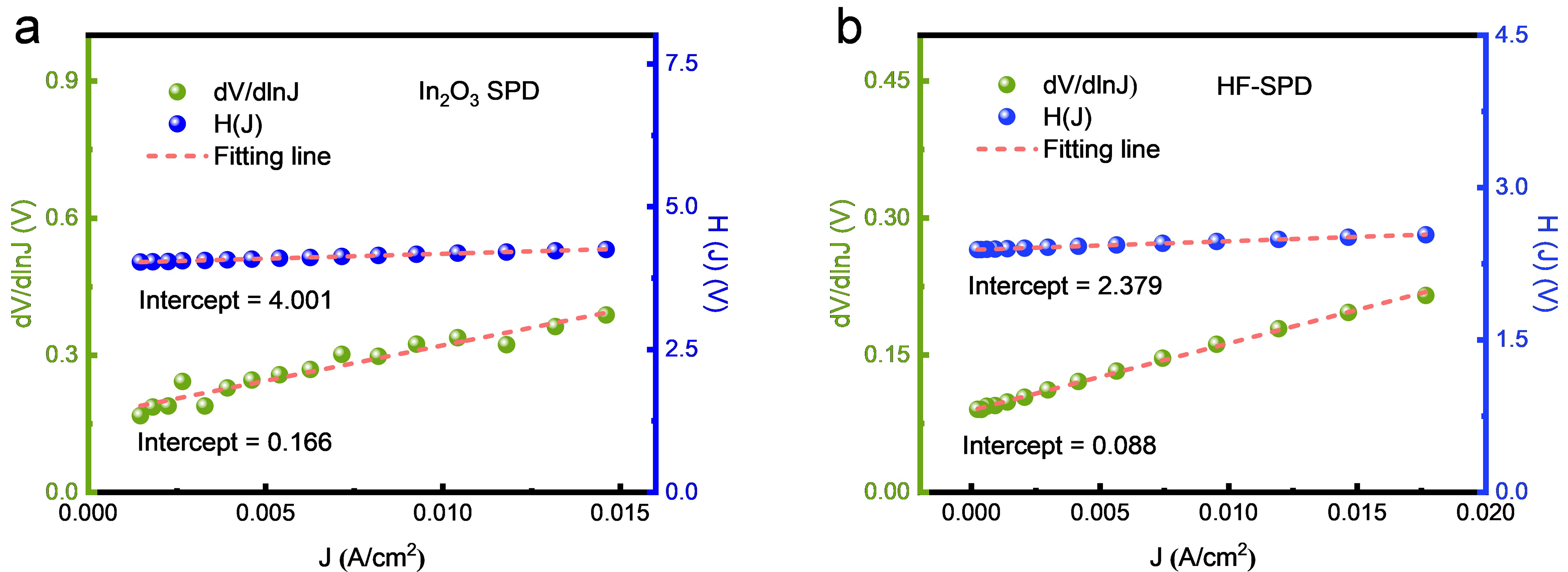


**Figure S10.** d*V*/dln*J* versus *J* and *H*(*J*) versus *J* curves of (a) the In_2_O_3_ SPD and (b) HF-SPD.


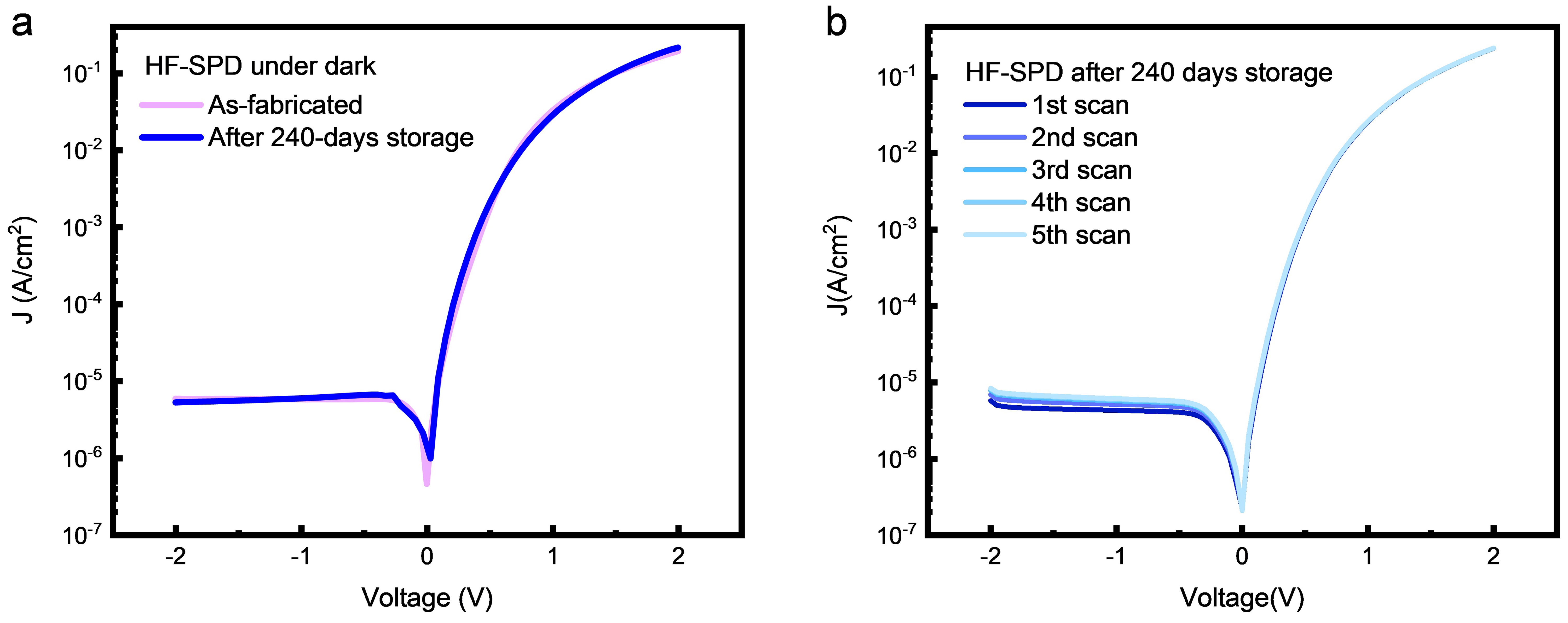


**Figure S11.** *J*-*V* characteristics of HF-SPDs in long-term ambient storage.


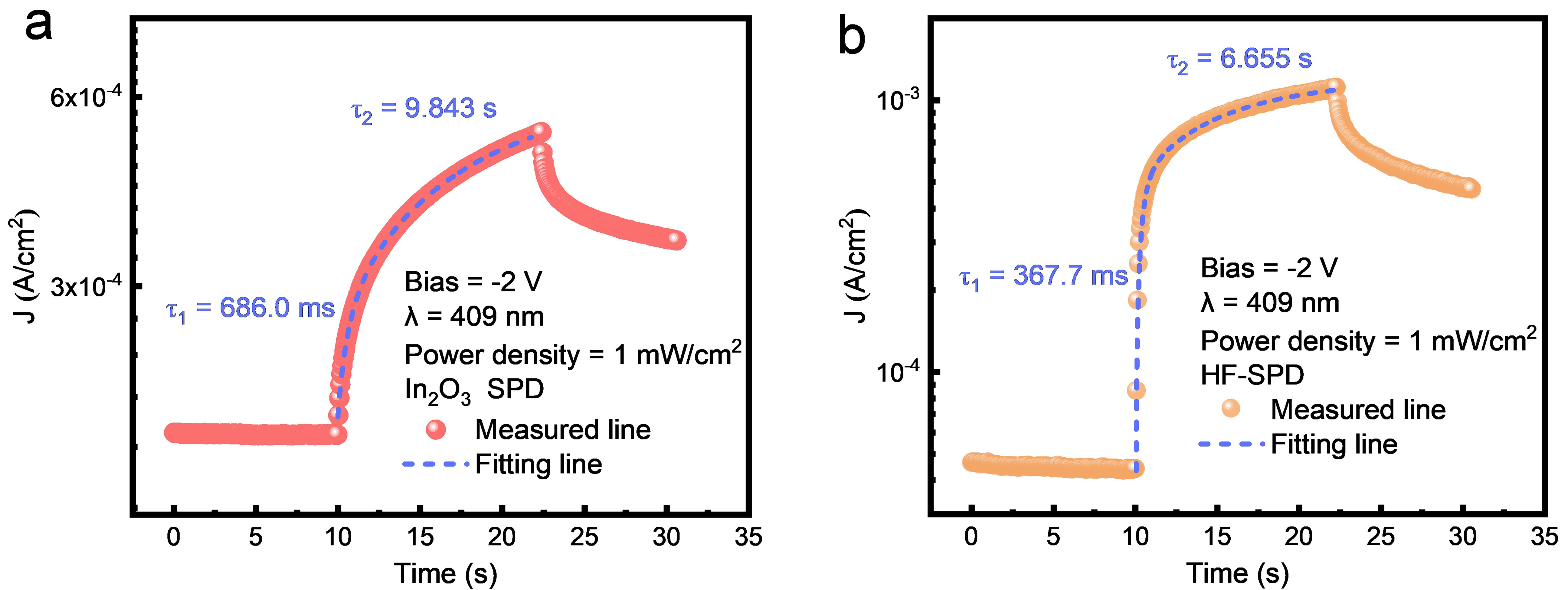


**Figure S12.** Dynamic responses of the In_2_O_3_ SPD and HF-SPD.


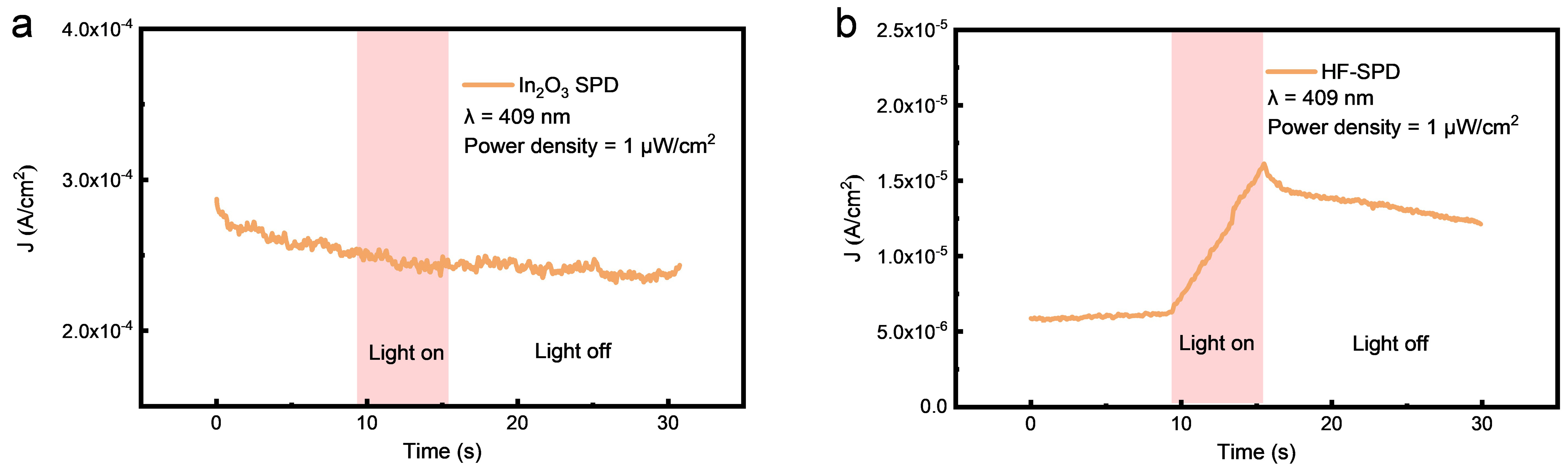


**Figure S13.** Minimum detection limits under weak illumination for the In_2_O_3_ SPD and HF-SPD.


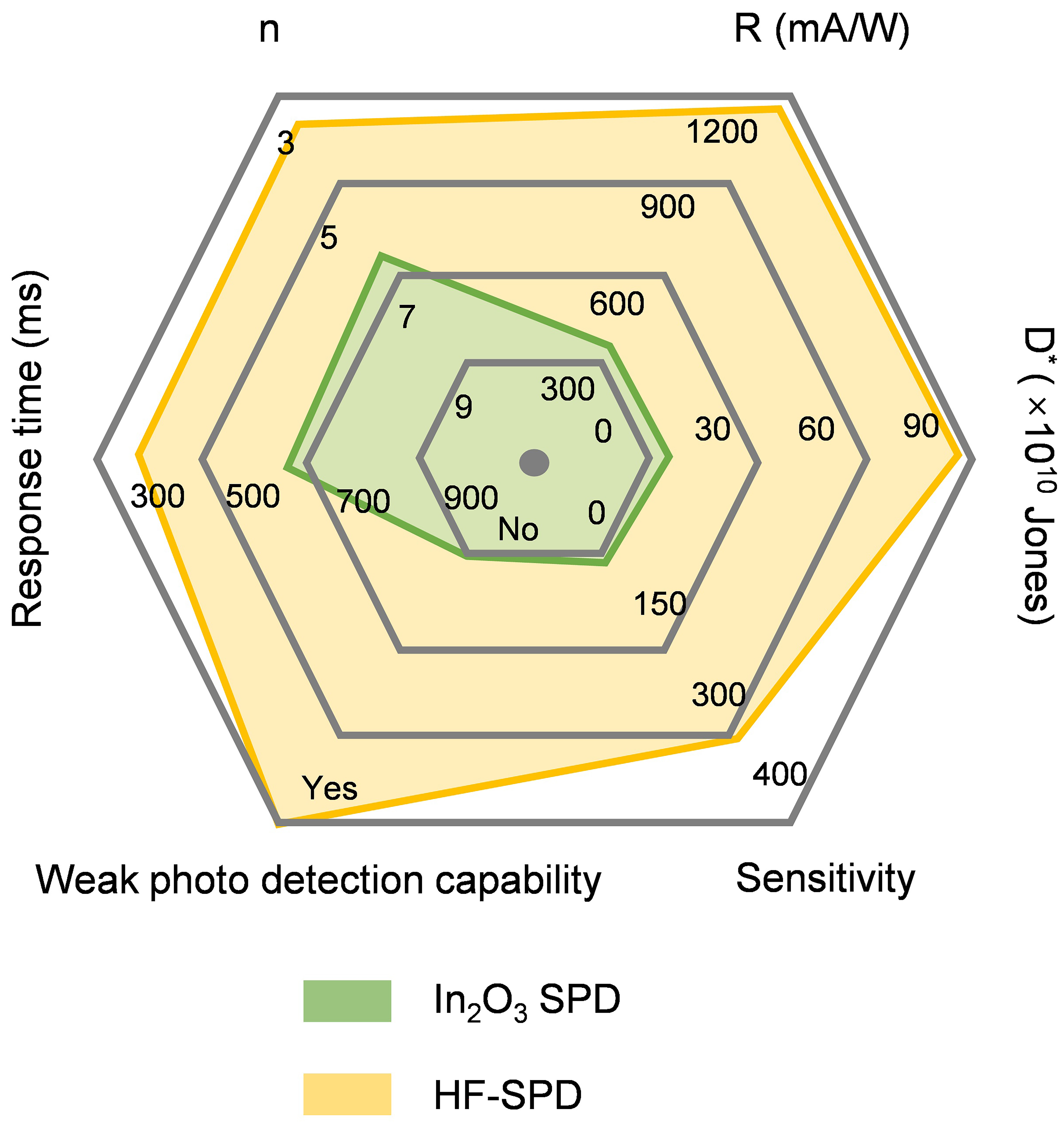


**Figure S14.** Comprehensive performance comparison between the In_2_O_3_ SPD and HF- SPD.


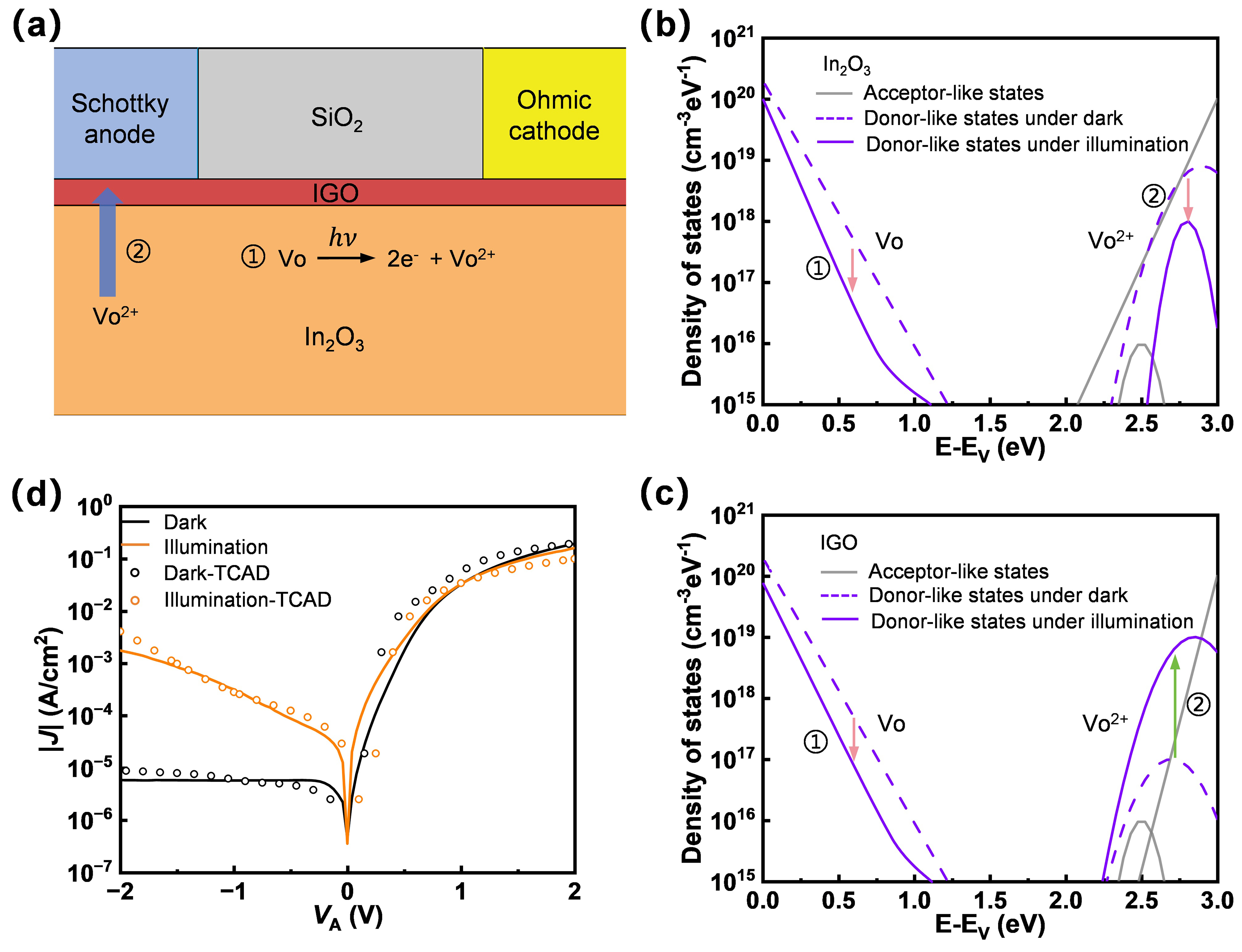


**Figure S15.** (a) Schematic structure of the simulated HF-SPD with corresponding DOS of (b) bulk and (c) interface. (d) The experimental and fitting *J*-*V* curves of HF-SPD under dark and illumination states.

Note: In oxide semiconductors, Vo and Vo^2+^ correspond to deep-level and shallow-level donor defects, respectively. Due to the illumination-induced transition of Vo into Vo^2+^, the Vo concentration decreases both in the bulk and at the surface. Driven by the external electric field, the charged Vo^2+^ species migrate from the bulk toward the surface and gradually accumulate in the depletion region, leading to an increasing Vo^2+^ concentration at the surface (Figure S14a and S14b). Under the dark state, the thermal emission current under reverse bias remains low and shows negligible dependence on the applied voltage. Under the illumination, the bias-accumulated Vo^2+^ at the Schottky interface facilitates the electron tunnelling, resulting in a pronounced photocurrent gain. According to the aforementioned ODS distributions and transport mechanisms in Figure 4a, the simulation results agree well with the experimental *J*-*V* characteristics.


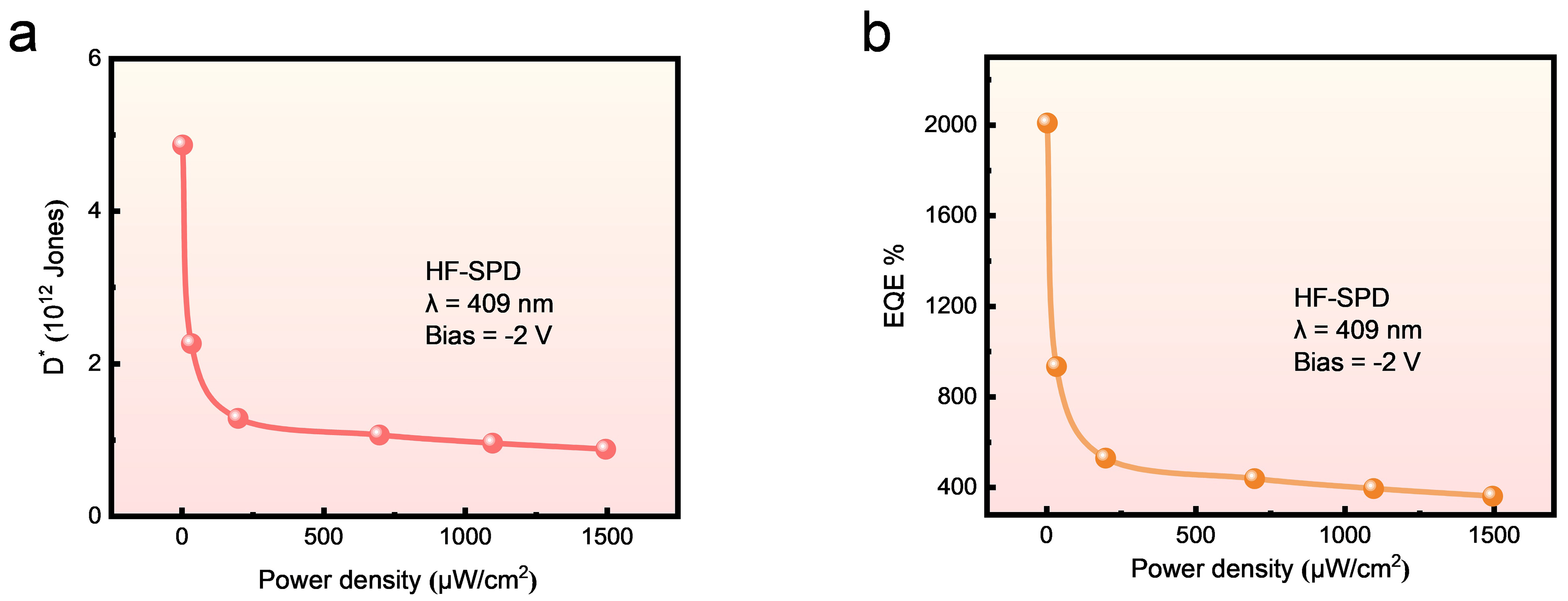


**Figure S16.** The dependences of *D*^*^ and EQE on the power density under the -2 V bias voltage.


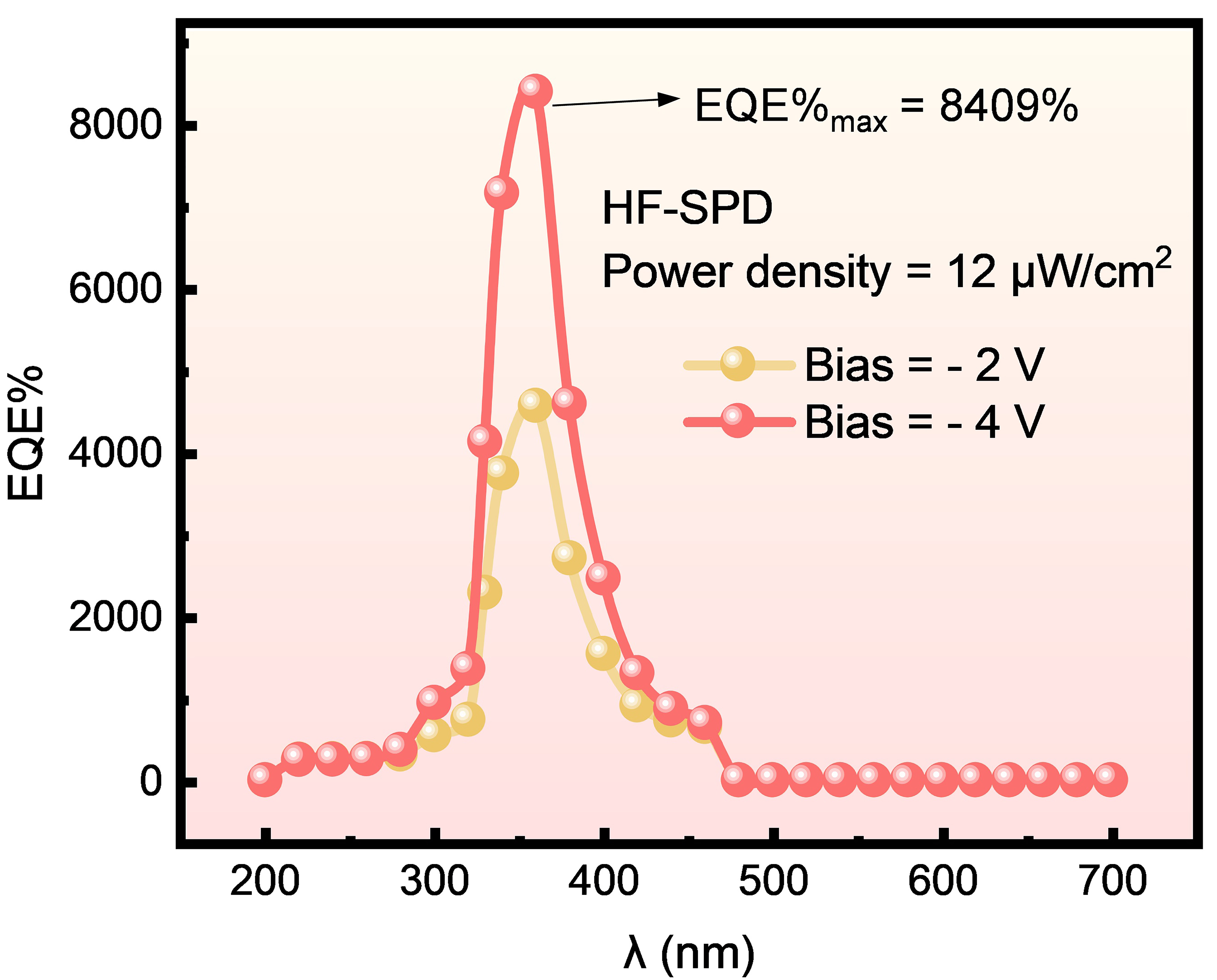


**Figure S17.** The relationships between EQE and the wavelength under -2 V and -4 V bias voltage.


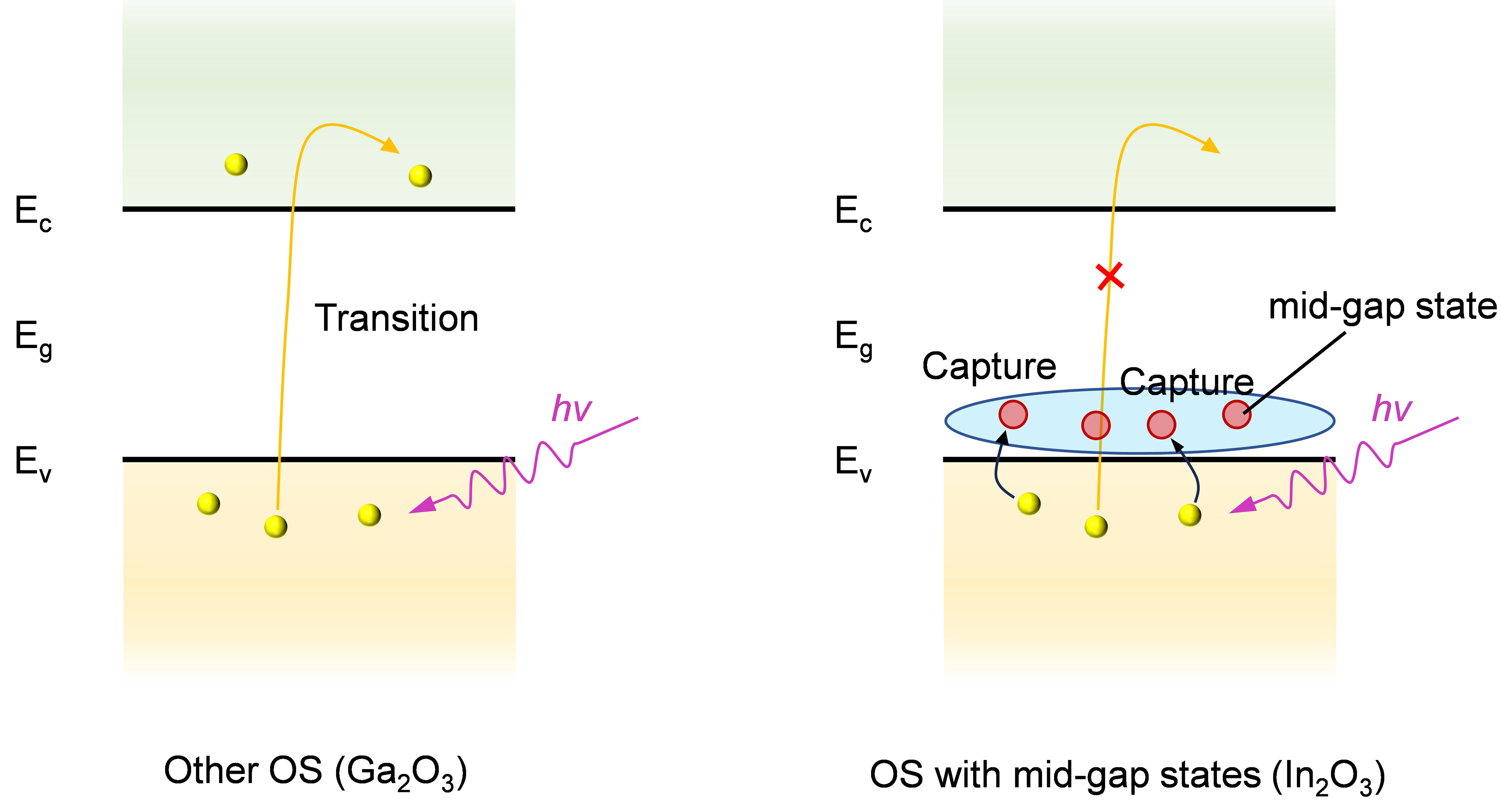


**Figure S18.** Schematic diagrams of the electron transitions from the valence band to the conduction band.


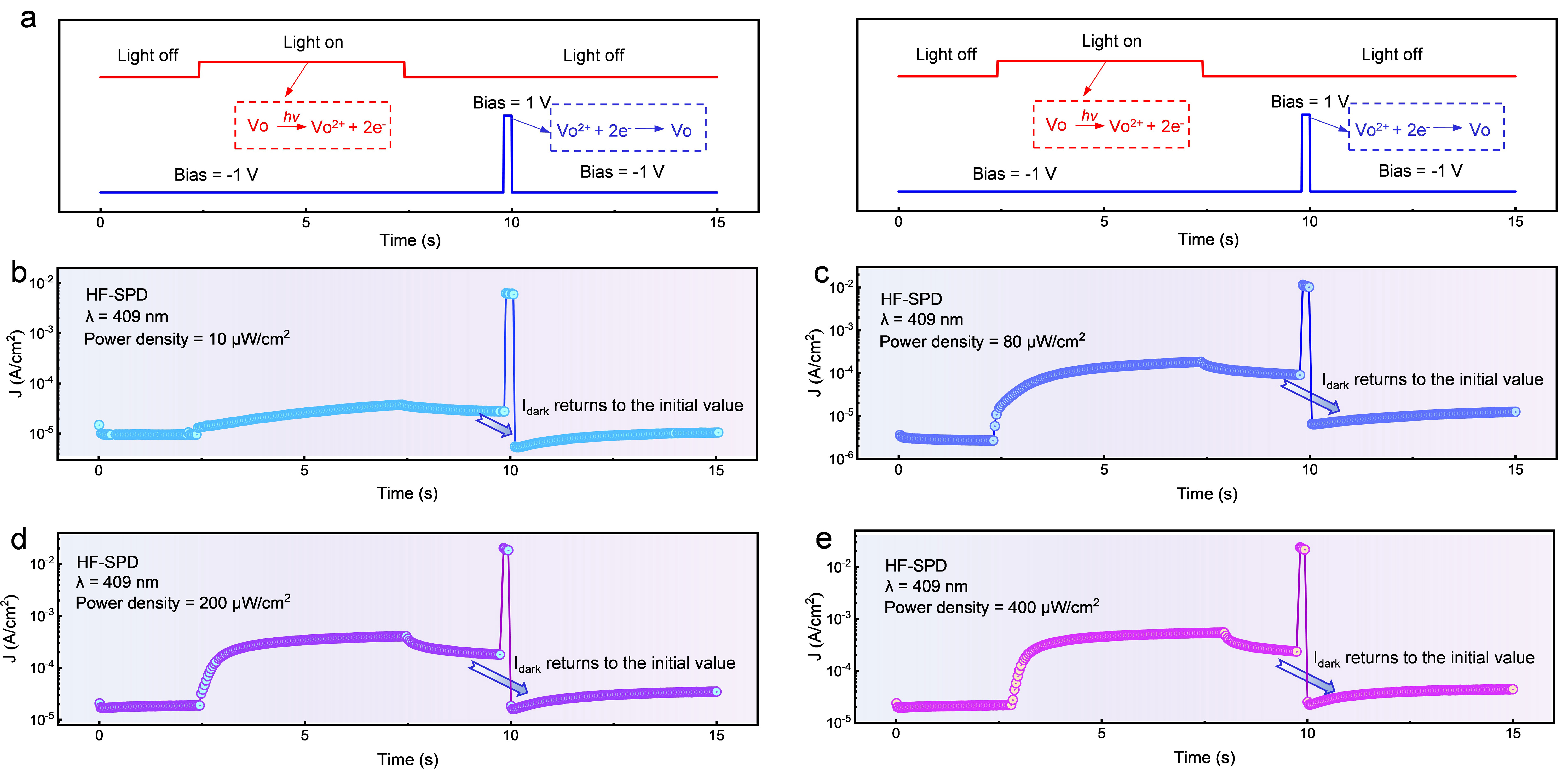


**Figure S19.** (a) Timing diagrams of optical pulses and electrical pulses. (b-e) The HF-SPD shows significant PPC under the illumination pulses with power densities of 10 μW/cm^2^, 80 μW/cm^2^, 200 μW/cm^2^ and 400 μW/cm^2^, respectively.


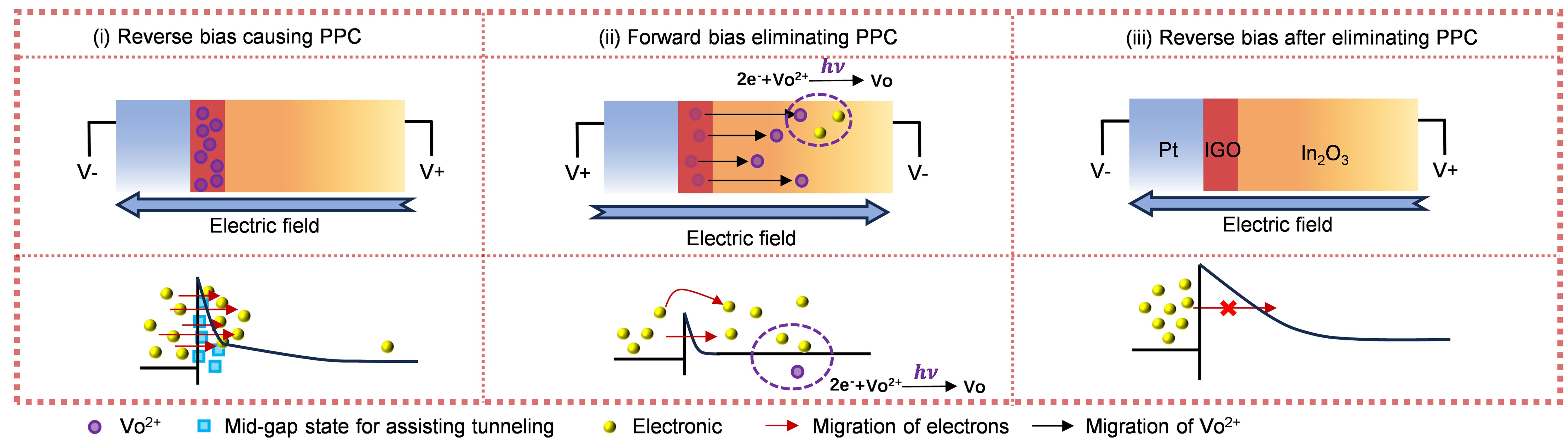


**Figure S20.** The generation and elimination mechanisms of PPC: (i) The long-lifetime Vo²⁺ defects in the low-electron-concentration depleted OS region maintain the high tunneling current through the Schottky barrier; (ii) Abundant electrons are emitted over the lowered Schottky barrier into OS and instantly recombine with Vo²⁺. (iii) The annihilation of interface mid-gap states eliminates the trap-assisted tunneling mechanism and re-elevates the Schottky barrier, recovering the low reverse-bias current.


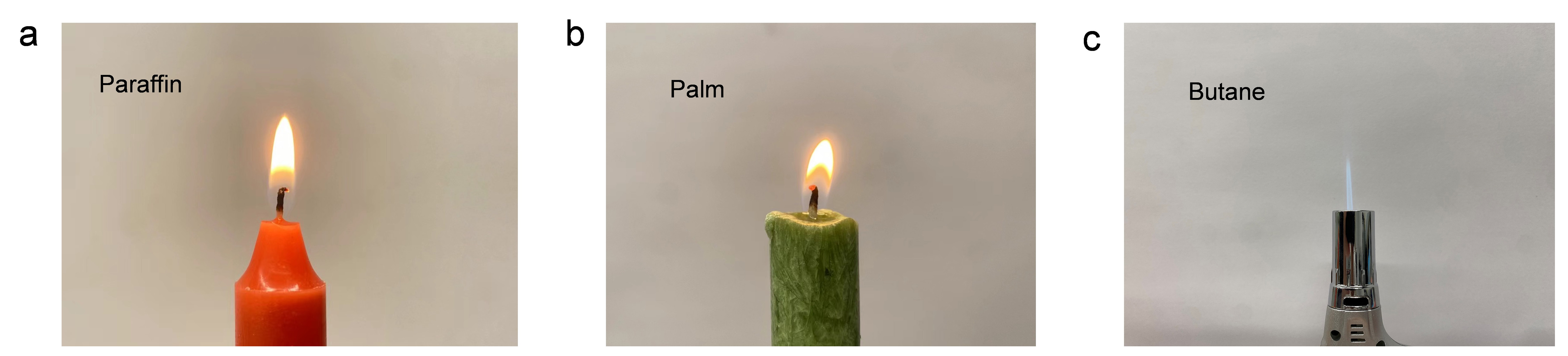


**Figure S21.** Photographs of flames produced by different combustion materials.


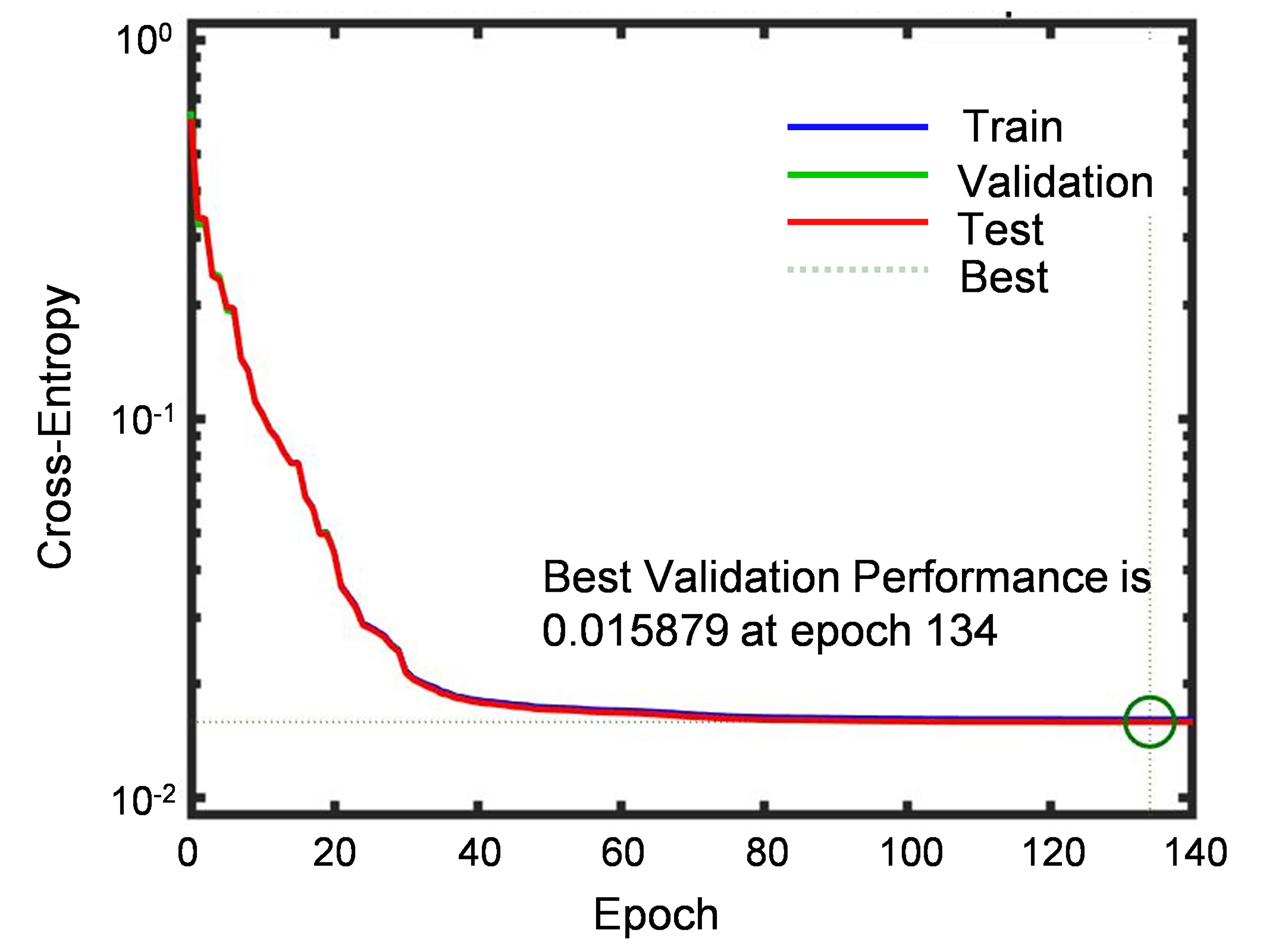


**Figure S22.** The Cross-Entropy of the MLP neural network attenuates and tends to stabilize with the increase of the number of epochs.

**Supplementary Table**

**Table S1.** Comparison of recently reported OS PDs and the proposed HF-SPD.

| **Material** | **Structure** | ***R* (A/W)** | ***D^*^* (Jones)** | **LT** | **Stability** | **Ref.** |
| --- | --- | --- | --- | --- | --- | --- |
| ZnO/ZnAl_2_O_4_ | PEC | 0.047 | 2.46×10^11^ | Yes | 30 days | [5] |
| LSMO/BTO/Ga_2_O_3_ | MSM | 1.1 | 1.2×10^12^ | No | \ | [6] |
| ZnO/Si | SPD | 0.341 | 7.72×10^13^ | Yes | \ | [7] |
| GaN/IGZO | PN junction | 0.26 | 1.90×10^13^ | No | \ | [8] |
| IGZO | MSM | 1.12 | 2×10^11^ | Yes | \ | [9] |
| CAL/IGZO | TFT | 1.57 | 8.47×10^11^ | Yes | 144 hours | [10] |
| Al/TiO_2_/p-Si | PN junction | 0.02 | 2.43×10^9^ | Yes | \ | [11] |
| In_2_O_3_ | PEC | 0.021 | 2.03×10^10^ | Yes | 90 days | [12] |
| In_2_O_3_ | PEC | 0.044 | 8.55×10^10^ | Yes | 270 days | [13] |
| ZnSnO_3_/In_2_O_3_ | MSM | 0.0007 | 8.30×10^9^ | Yes | \ | [14] |
| HfO_2_/IGO/In_2_O_3_ | SPD | 27.75 | 2.036×10^13^ | Yes | 240 days | HF-SPD |

**Reference**

[1] J. Park, S. Yang, C. Choi, S. Choi, C. Kim, *ACS Appl. Mater. Interfaces* **2021**, *13*, 22828.

[2] C. V. Prasad, J. H. Park, J. Y. Min, W. Song, M. Labed, Y. Jung, S. Kyoung, S. Kim, N. Sengouga, Y. S. Rim, *Mater. Today Phys.* **2023**, *30*, 100932.

[3] T. Huang, Y. Zhang, J. Li, Y. Zhang, L. Lu, H. Zhou, S. Zhang, *IEEE Electron Device Lett.* **2025**, *46*, 1123.

[4] D. Ho, S. Choi, H. Kang, B. Park, M. N. Le, S. K. Park, M. G. Kim, C. Kim, A. Facchetti, *ACS Appl. Mater. Interfaces* **2023**, *15*, 33751.

[5] H. Wu, L. Shu, Q. Zhang, S. Sha, Z. Liu, S. Li, S. Yan, W. Tang, Y. Wang, Z. Wu, K. Lin, Q. Li, J. Miao, X. Xing, *Adv. Mater.* **2025**, *37*, 2412717.

[6] S. Sun, W. Li, Y. Zhang, Q. Gao, N. Zhang, Y. Qin, W. Feng, *Small* **2025**, *12*, 2407107.

[7] Y. Liao, Y. J. Kim, M. Kim, *Chem. Eng. J.* **2023**, *476*, 146838.

[8] Y. Zhang, X. Feng, F. Yang, Y. Duan, Y. Zhao, T. Chen, P. Wang, J. Guo, Z. Du, G. Cheng, *Nano Res.* **2025**, *18*, 94907007.

[9] R. Kishore, K. Vishwakarma, A. Datta, *IEEE J. Quantum Electron.* **2023**, *59*, 4000107.

[10] H. Son, D. H. Choi, K. Park, J. Chung, B. H. Kang, H. J. Kim, *ACS Appl. Mater. Interfaces* **2024**, *16*, 67909.

[11] D. E. Yıldız, A. Kocyigit, M. Yıldırım, *Opt. Mater.* **2023**, *145,* 114371.

[12] M. Q. Cui, Z. Shao, L. H. Qu, X. Liu, H. Yu, Y. Wang, Y. Zhang, Z. Fu, Y. Huang, W. Feng, *ACS Appl. Mater. Interfaces* **2022**, *14*, 39046.

[13] N. Zhang, M. Cui, J. Zhou, Z. Shao, X. Gao, J. Liu, R. Sun, Y. Zhang, W. Li, X. Li, J. Yao, F. Gao, W. Feng, *ACS Appl. Mater. Interfaces* **2024**, *16*, 19167.

[14] S. Veeralingam, S. Badhulika, *Nano Energy* **2022**, *98*, 107354.
